# Supplementary material for: Stretchable surface electromyography electrode array patch for tendon location and muscle injury prevention
Source: Nat Commun. 2023 Oct 14;14:6494. doi: 10.1038/s41467-023-42149-x (PMC10576757; doi:10.1038/s41467-023-42149-x)
Supplement: Supplementary file 1 — Supplementary information [file 41467_2023_42149_MOESM1_ESM.pdf]

Supplementary materials

Stretchable surface electromyography electrode array patch for tendon location and muscle injury prevention

Shuaijian Yang[1,2], Jinhao Cheng[1], Jin Shang[1], Chen Hang[1], Jie Qi[1], Leni Zhong[1], Qingyan Rao[1], Lei He[1], Chenqi Liu[1], Li Ding[1], Mingming Zhang[1], Samit Chakrabarty[2], Xingyu Jiang[1]**

1 Shenzhen Key Laboratory of Smart Healthcare Engineering, Guangdong Provincial Key Laboratory of Advanced Biomaterials, Department of Biomedical Engineering, Southern University of Science and Technology, Shenzhen, Guangdong 518055, P. R. China.
2 School of Biomedical Sciences, Faculty of Biological Sciences, University of Leeds, Leeds, LS2 9JT, United Kingdom.

* Corresponding author, E-mail: jiang@sustech.edu.cn; S.Chakrabarty@leeds.ac.uk

Supplementary Text 1 Normalization of mean frequencies.

Take mean frequencies recorded by the first column as an example,

$$m_1, m_5, m_9, m_{13}, m_{17}, m_{21}.$$

According to the discussion with Fig. 7c, we found they are in descending order that

$$m_1 > m_5 > m_9 > m_{13} > m_{17} > m_{21}.$$

Then the normalization can be expressed as

$$n_1 = 1, n_5 = \frac{m_5 - m_{21}}{m_1 - m_{21}}, n_9 = \frac{m_9 - m_{21}}{m_1 - m_{21}}, n_{13} = \frac{m_{13} - m_{21}}{m_1 - m_{21}}, n_{17} = \frac{m_{17} - m_{21}}{m_1 - m_{21}}, n_{21} = 0,$$

When $n_i > 0.5$, the channel i was considered on tendon part; when $n_i = 0.5$, the channel i was considered on muscle-tendon junction; when $n_i < 0.5$, the channel i was considered on muscle part. Usually, 0.5 is between n_i and n_{i+1}, while a linear fitting is used to determine the junction location between the two certain channels.

Supplementary Text 2 The effect of inter-electrode distance change on muscle-tendon junction location.

In muscle-tendon junction location of biceps distal tendon, we measured the IEDs between adjacent channels.

Flexion degree	$IED_{21\text{-}17}$ (mm)	$IED_{17\text{-}13}$ (mm)	$IED_{13\text{-}9}$ (mm)	$IED_{9\text{-}5}$ (mm)	$IED_{5\text{-}1}$ (mm)
30°	15 mm	15 mm	15 mm	15 mm	15 mm
0°	15 mm	15 mm	16 mm	18 mm	18 mm
110°	15 mm	15 mm	14 mm	13 mm	12 mm

Because the TPP electrodes are adhesive which can attach on the skin firmly, so the IED change is the same as the skin deformation between two adjacent electrodes. We measured the IEDs in three different states for the arm which are full extension (0°), full flexion (110°) and relax (30°). The array was attached on the skin during the relax of the arm, so the IEDs are all 15 mm. We found the on the muscle part, the deformations of skin are not obvious even the muscle is moving.

For example, when the junction is right middle between channel 5 and 1 when full extension, the calculated distance between junction and channel 21 is $D_{ce} = IED_{21\text{-}17}(30°)+IED_{17\text{-}13}(30°)+IED_{13\text{-}9}(30°)+IED_{9\text{-}5}(30°)+1/2\ IED_{5\text{-}1}(30°)$;

the realistic distance between junction and channel 21 is $D_{re} = IED_{21\text{-}17}(0°)+IED_{17\text{-}13}(0°)+IED_{13\text{-}9}(0°)+IED_{9\text{-}5}(0°)+1/2\ IED_{5\text{-}1}(0°)$。

When the junction is right middle between channel 13 and 17 when full flexion,

the calculated distance between junction and channel 21 is $D_{cf} = IED_{21\text{-}17}(30°)+1/2\ IED_{17\text{-}13}(30°)$;

the realistic distance between junction and channel 21 is $D_{rf} = IED_{21\text{-}17}(110°)+1/2\ IED_{17\text{-}13}(110°)$.

Considering the absolute displacement D_a of channel 21 between flexion and extension in the space,

then the calculated displacement is $D_{ce}-D_{cf}+D_a = 1/2\ IED_{17\text{-}13}(30°)+IED_{13\text{-}9}(30°)+IED_{9\text{-}5}(30°)+1/2\ IED_{5\text{-}1}(30°) + D_a$;

the realistic displacement is $D_{re}-D_{rf}+D_a = 1/2\ IED_{17\text{-}13}(0°)+IED_{13\text{-}9}(0°)+IED_{9\text{-}5}(0°)+1/2\ IED_{5\text{-}1}(0°)+D_a$;

The reason why we choose distance between junction and channel 21 instead of channel 1 is that, we found $D_a(21) \approx 0$ mm, while $D_a(1) \approx 40$ mm. So calculating from the muscle end can lower the effect of huge skin deformation.

Finally, the calculated displacement is 45 mm and the realistic displacement is 50.5 mm. The error is about 10% which should be considered as acceptable.

For the Achilles tendon identification, we didn't observe obvious skin deformation between flexion and extension, so we choose not to consider this factor in that application.

Supplementary Text 3 Definitions of RMS, median frequencies and mean frequencies.

$$RMS = \sqrt{\frac{1}{N}\sum_{i=1}^{N} x_i^2}$$

$$MNF = \sum_{j=1}^{M} f_j P_j \bigg/ \sum_{j=1}^{M} P_j$$

$$\sum_{j=1}^{MDF} P_j = \sum_{j=MDF}^{M} P_j = \frac{1}{2}\sum_{j=1}^{M} P_j$$

where x_i is voltage amplitude of EMG signal at the data point i; N is the number of data points; f_j is the frequency value of EMG power spectrum at the frequency bin j; P_j is the EMG power spectrum at the frequency bin j; and M is the length of frequency bin[31].

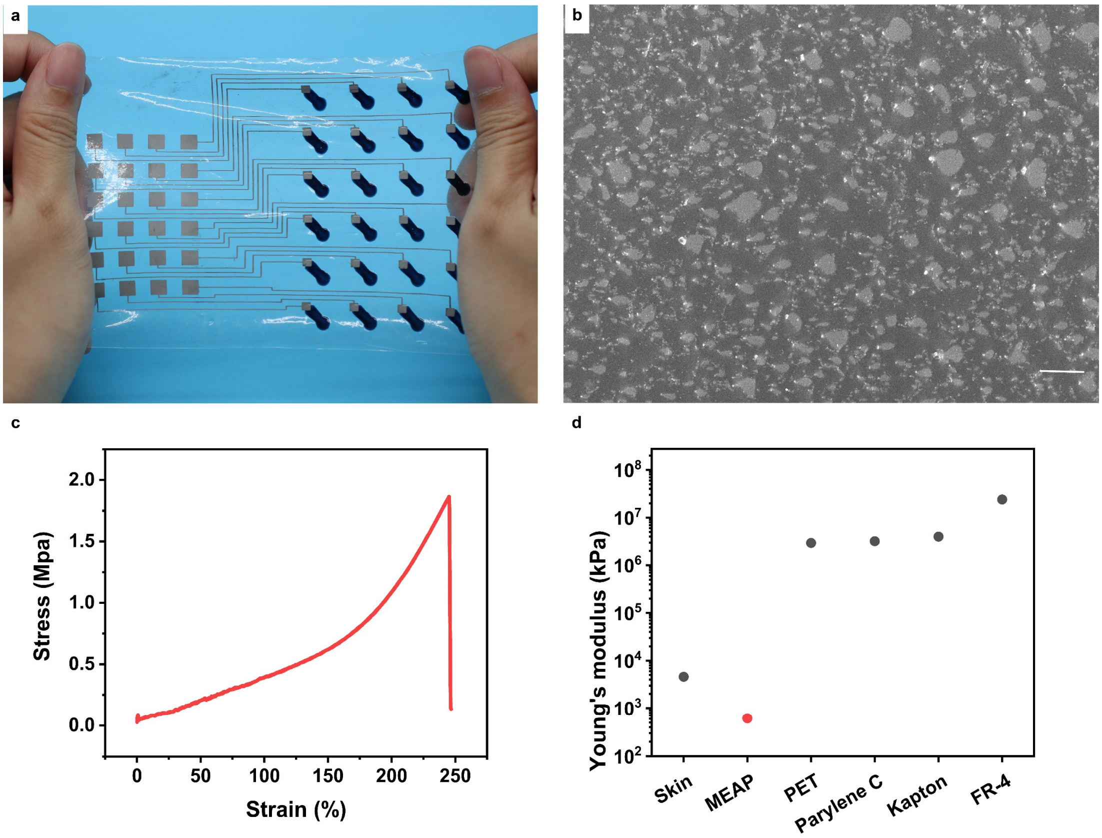

Supplementary Fig. 1 Characterizations of the MEAP.

a Photograph of the MEAP.

b SEM micrograph of the MPC circuit. Scale bar: 10 µm. Micrographs (x3) were collected independently with similar results.

c Stress-strain curves of the substrate of MEAP.

d Comparison of Young's modulus between common substrates of sEMG electrodes[1].

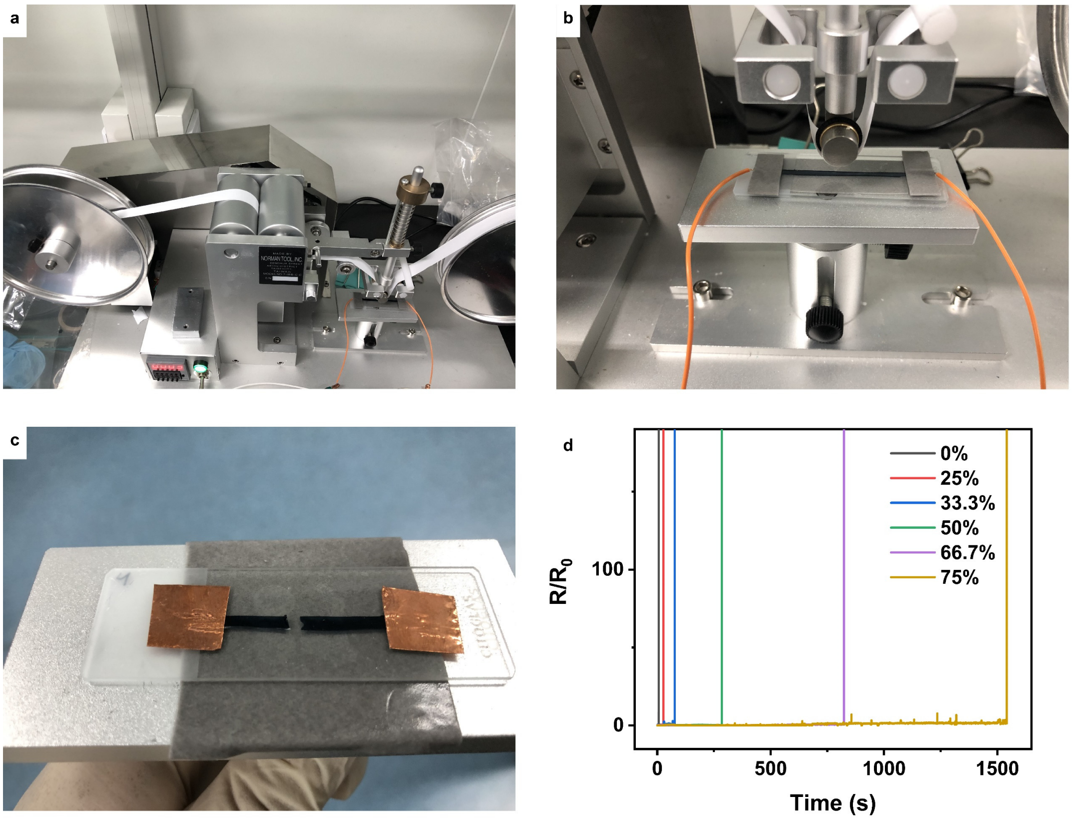

Supplementary Fig. 2 Abrasion-resistant test of the PEDOT:PSS-PVA strip.

a, b Photographs of the RCA abrasion wear tester and PEDOT:PSS-PVA strip. Two ends of the strip were connected to electrochemical workstation to monitor resistances.

c A photograph of PEDOT:PSS-PVA strip after cracking.

d Resistance changes of strips with different PVA addition during the test. With more PVA addition, the film can stand longer time to abrasion.

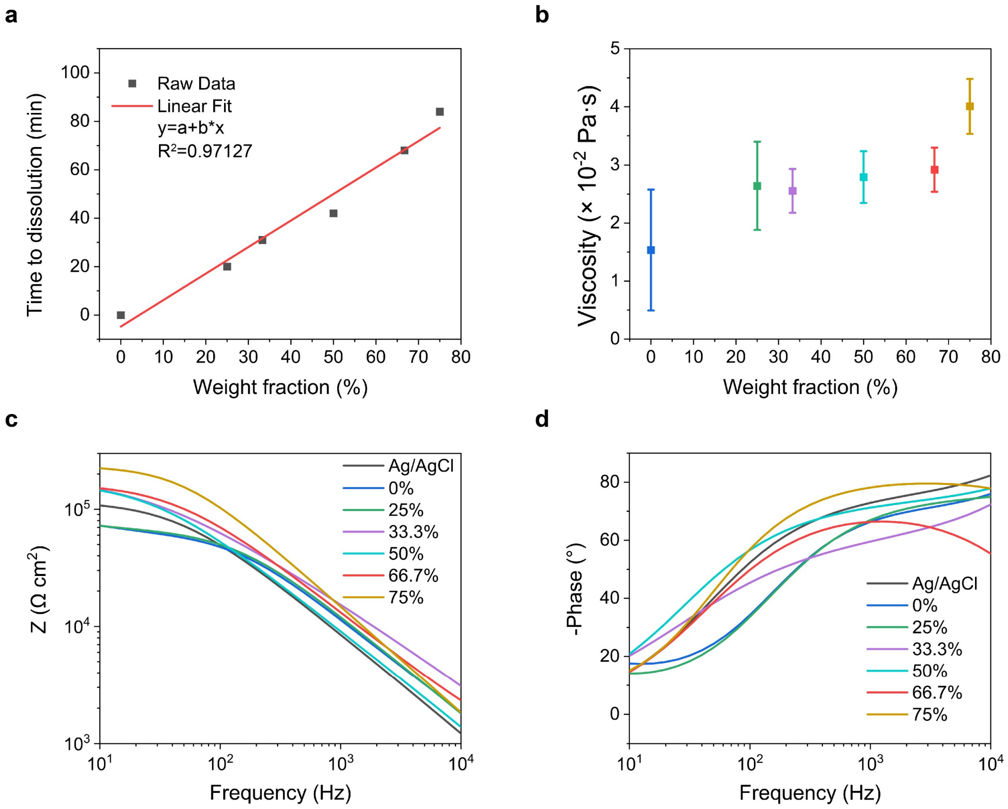

Supplementary Fig. 3 Characterizations of PEDOT:PSS-PVA electrodes with different PVA additions.

a Water-resistance of PEDOT-PVA electrodes. The water resistance of PEDOT-PVA film was tested in a water bath of sonicator.

b The viscosities of PEDOT-PVA solutions. Lower viscosity is preferred for easier patterning of the solution. n=3 samples for each measurement.

c, d The impedances and phases of the Ag/AgCl electrode and PEDOT-PVA electrodes on the skin.

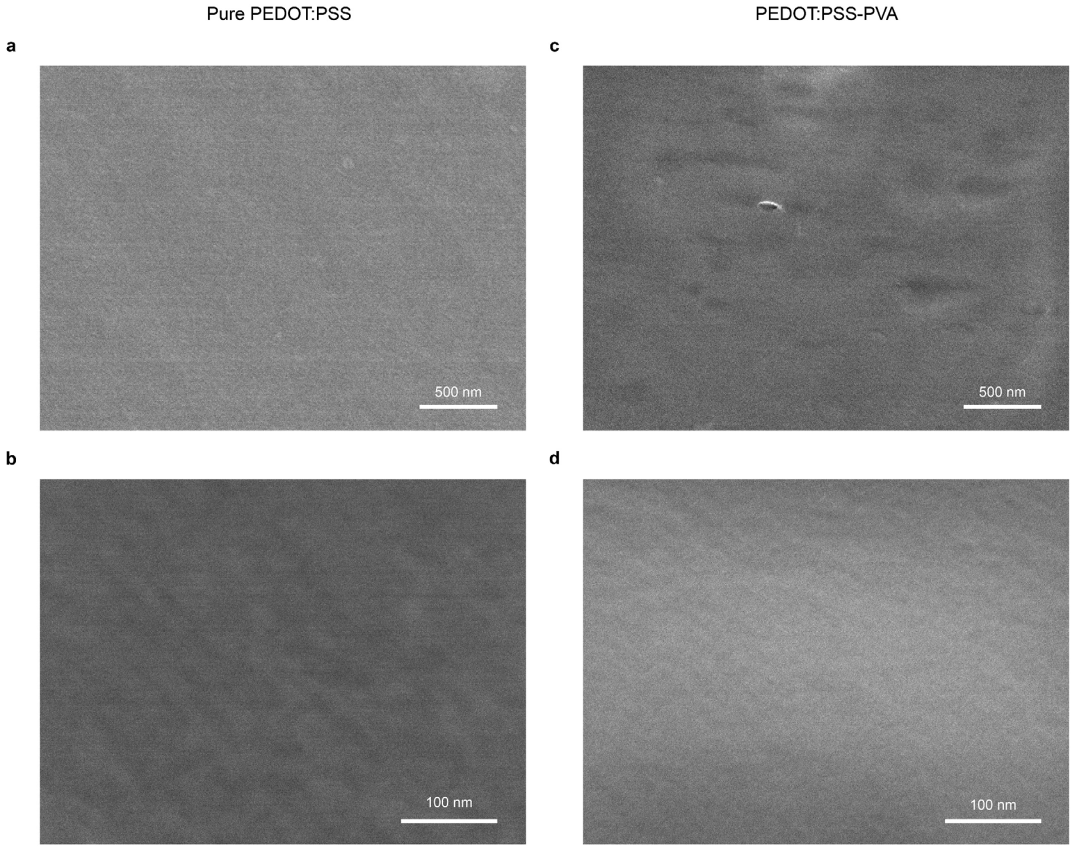

Supplementary Fig. 4 SEM images of the PEDOT:PSS film with or without PVA addition.

a, b SEM images of the electrode film made by pure PEDOT:PSS.

c, d SEM images of the electrode film made by PEDOT:PSS with 66.7 wt% of PVA and 5 vol% DMSO. In comparison with **b** and **d**, the PVA can be mixed with PEDOT:PSS and build up much more uniform film than pure PEDOT:PSS films. The good miscibility can be ascribed to the strong interaction between PVA and PSSH by hydrogen bonds.

Micrographs (x3) were collected independently with similar results.

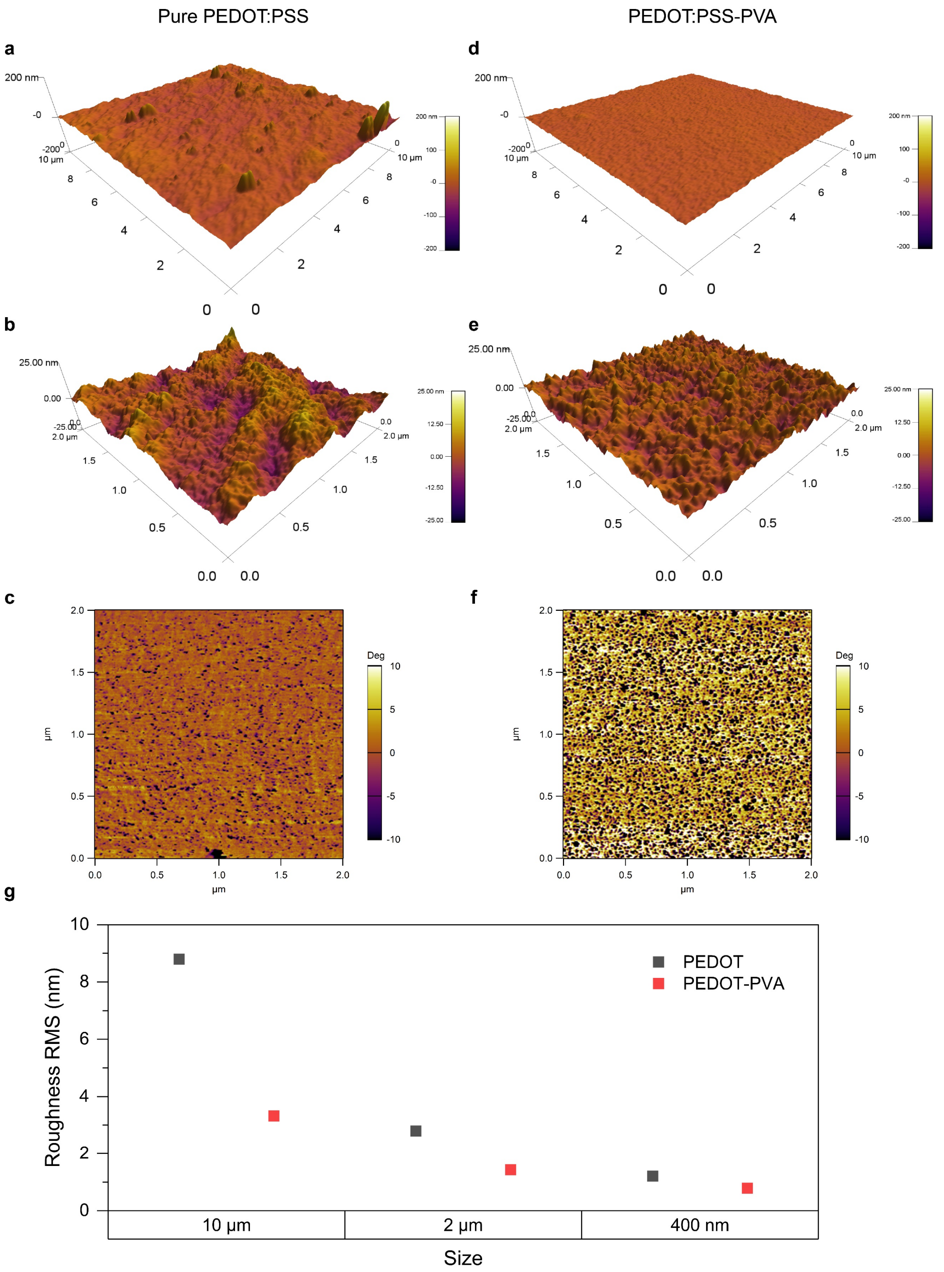

Supplementary Fig. 5 AFM images of the PEDOT:PSS film with or without PVA addition.

a-c AFM images and phase image of the electrode film made by pure PEDOT:PSS.

d-f AFM images and phase image of the electrode film made by PEDOT:PSS with 66.7 wt% of PVA and 5 vol% DMSO. Results of AFM images verified that PVA addition can smooth electrode films. Results of phase images show PVA addition introduce new phase into the film compared to pure PEDOT:PSS film, and phases have good miscibility in the film.

g Surface roughness of two types of PEDOT:PSS films measured using AFM. PEDOT and PEDOT-PVA are abbreviations of pure PEDOT:PSS and PEDOT:PSS with 66.7 wt% of PVA, respectively. PEDOT-PVA film consists of smaller grains and indicates smoother surface than the pure PEDOT:PSS film, which are responsible for the higher flexibility[2].

Micrographs (x3) were collected independently with similar results.

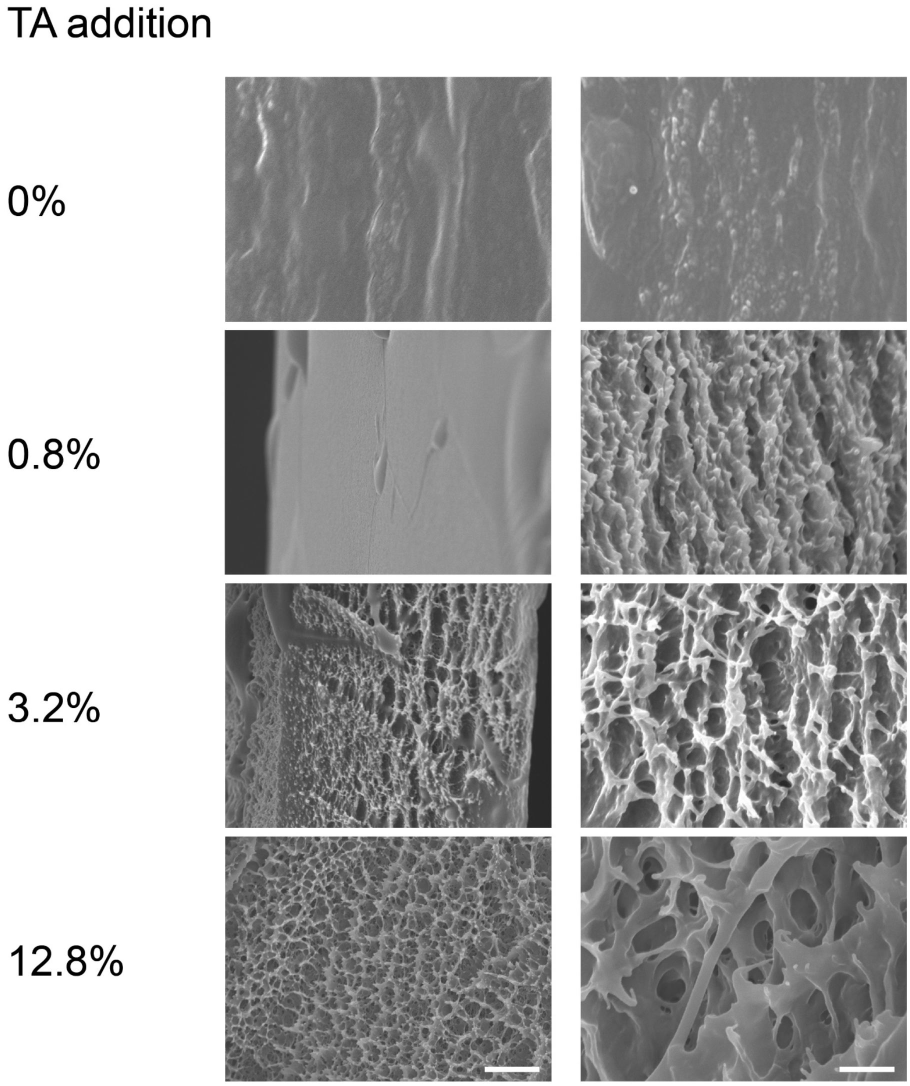

Supplementary Fig. 6 The cross-sectional SEM images of TPP films with different TA additions.

Results showed with increasing TA addition, the film turned from a dense film to a porous structure, making it gradually transform to hydrogel. The scale bars in images are 4 and 1 μm, respectively. Micrographs (x3) were collected independently with similar results.

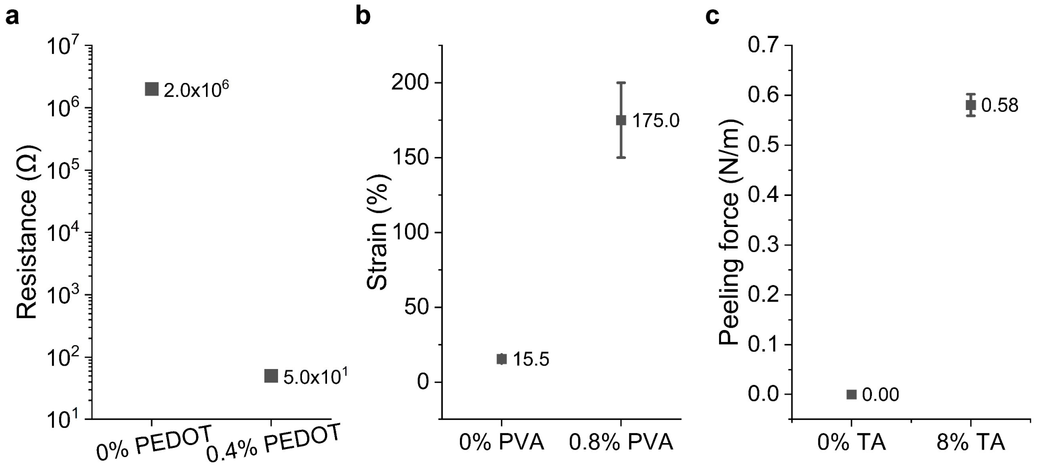

Supplementary Fig. 7 The verification of each constituent's indispensability in TPP film. a The change of resistance with or without PEDOT:PSS in TPP film. **b** The change of strain with or without PVA in TPP film. **c** The change of strain with or without TA in TPP film.

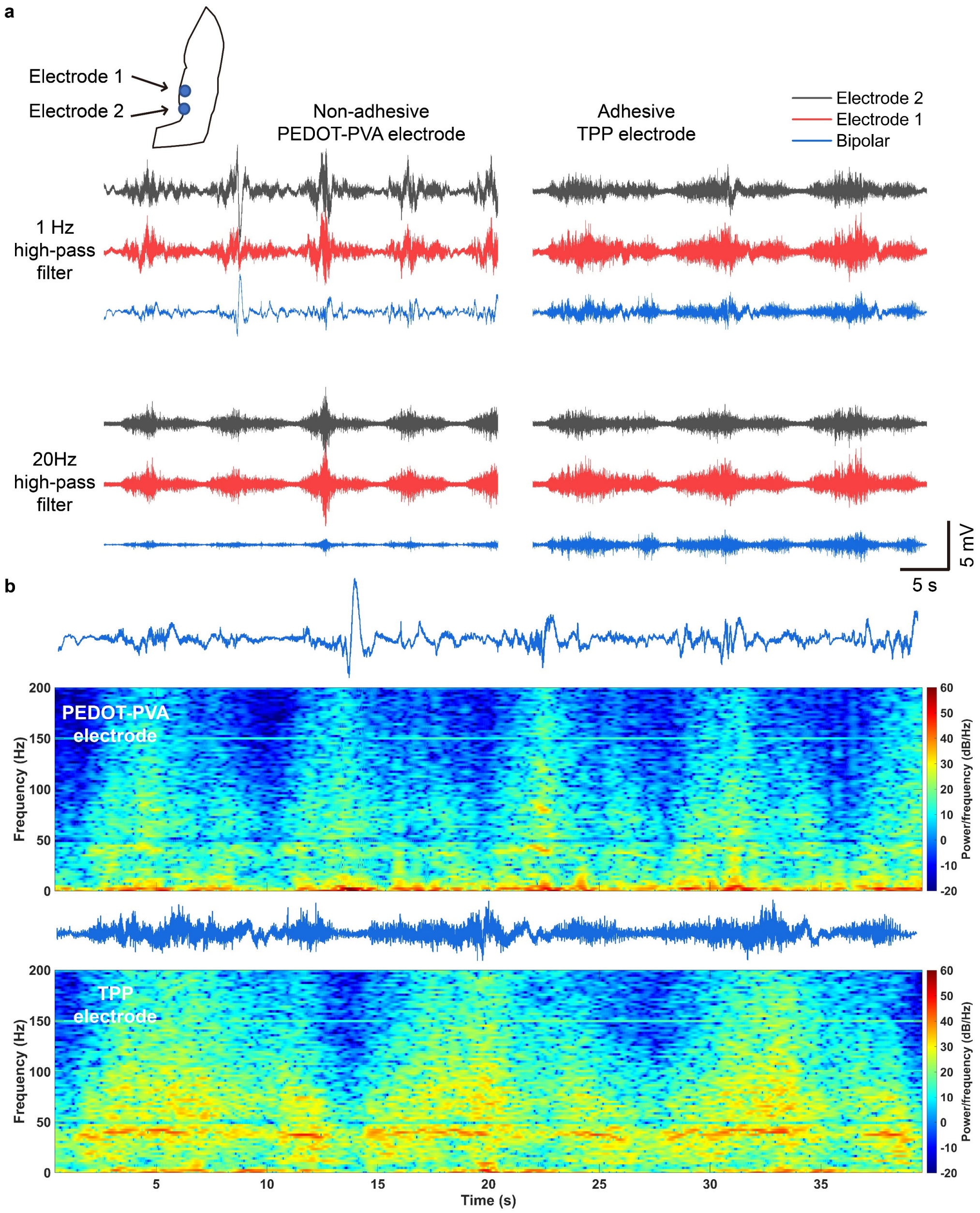

Supplementary Fig. 8 Recording performance by electrodes with or without adhesiveness during dynamic task.

a sEMG results filtered by 1 Hz and 20 Hz high-pass filter. Signals recorded by PEDOT-PVA electrodes showed severe instability with 1 Hz high-pass filter because the non-adhesiveness cause relative movement between electrodes and skin, causing motion artifacts in low frequency domain. By contrast, the adhesive TPP electrodes give much more stable recording in such circumstance.

b Spectrogram comparison between results recorded by two types of electrodes. Activation of EMG signals at 40 Hz was clear to see in results recorded by TPP electrodes while it was hard to tell with PEDOT-PVA electrodes. These show the advantage of adhesive TPP electrodes.

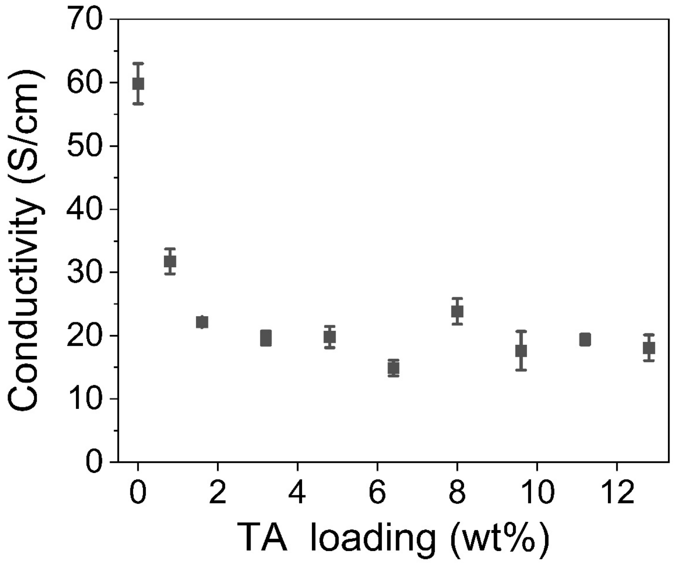

Supplementary Fig. 9 Conductivity of TPP films with different TA loading. n=3 samples for each measurement.

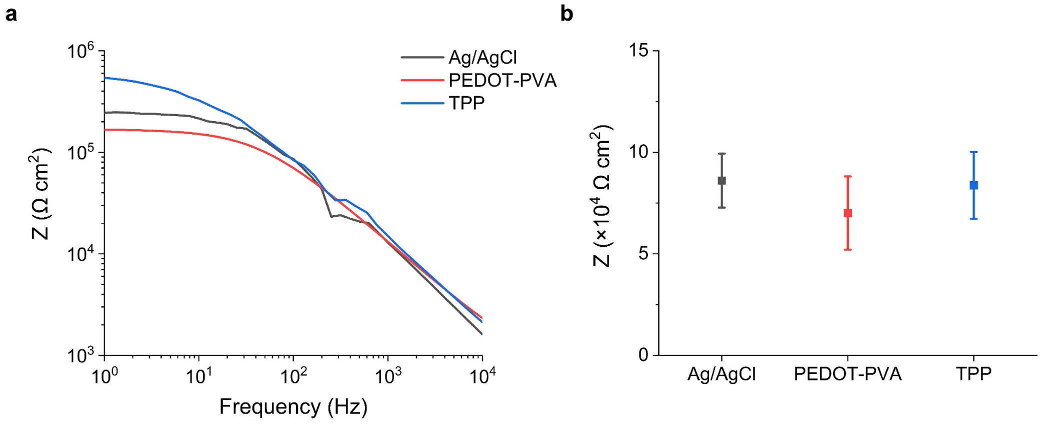

Supplementary Fig. 10 Comparisons of impedance between different types of electrodes.

a Bode plots of three electrodes tested on the skin. Specially for sEMG, the impedances from 20 – 200 Hz should be focused on.

b The impedances of three electrodes measured at 100 Hz. n=3 samples for each measurement.

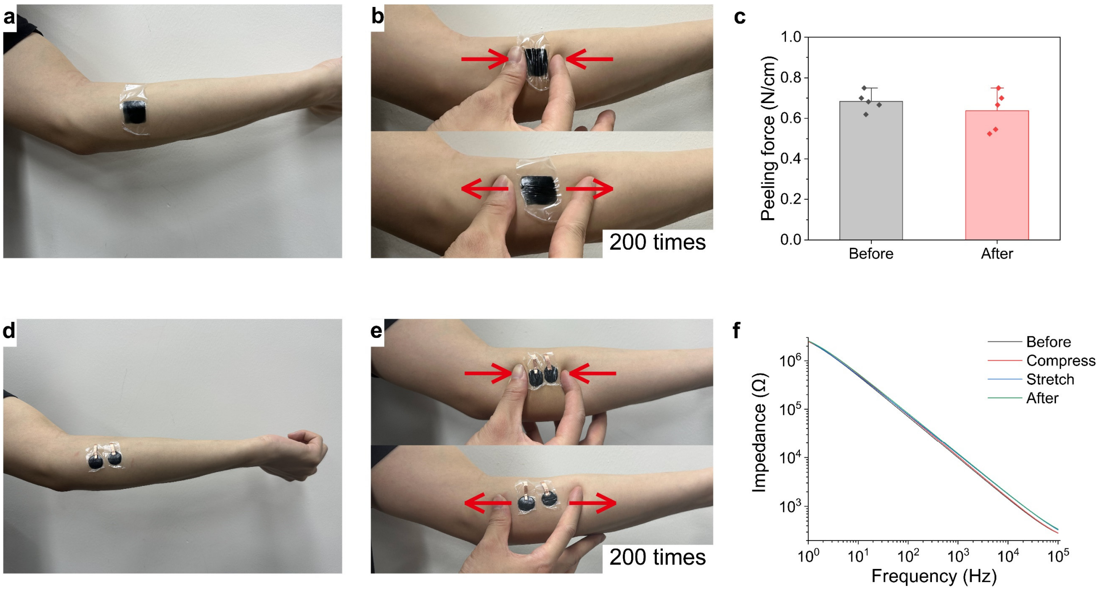

Supplementary Fig. 11 The change in adherence and impedance of TPP electrodes on the skin.

a, b images of the electrode applied to the skin and the motions made during the adhesion test.

c The peeling force of TPP electrodes off the skin before and after motions. n=5 samples for each measurement.

d, e Images of the electrode applied on the skin and the process of movements during impedance test.

f The impedance of TPP electrodes on the skin before, after motions and in the state of compressing and stretching.

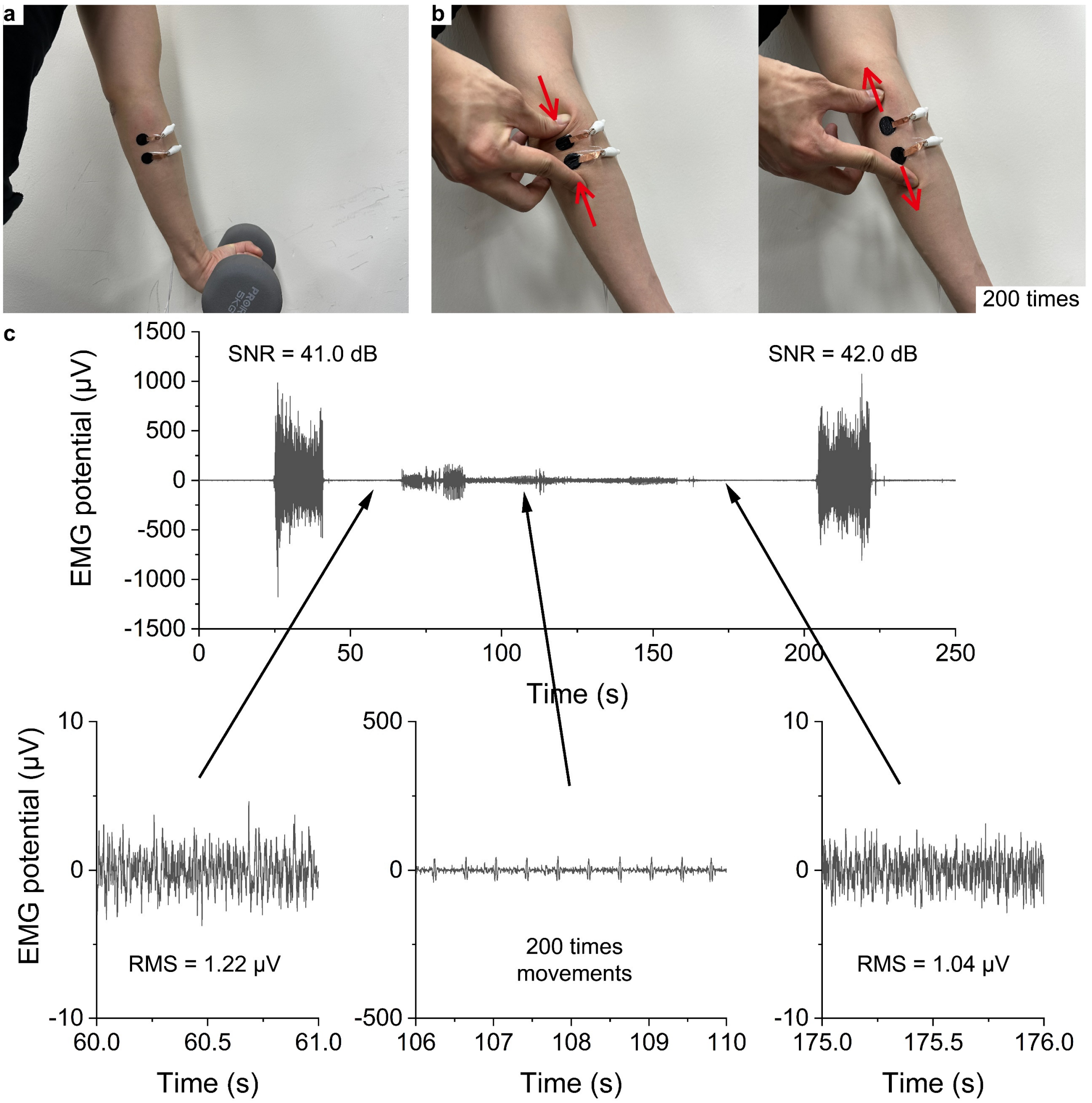

Supplementary Fig. 12 SNR variation and baseline noise levels of TPP electrodes on the skin. **a, b** Images showing the electrode applied to the skin and the motions made to compare SNR. **c** Demonstration of the entire process of recording using the TPP electrodes applied on skin before and after motions. The baseline noise level was reduced to 1.04 μV from 1.22 μV which was stable even after the motions. As a result, there was little change in the SNR of signals, showing the stability of TPP electrodes on the skin even after compression or stretching.

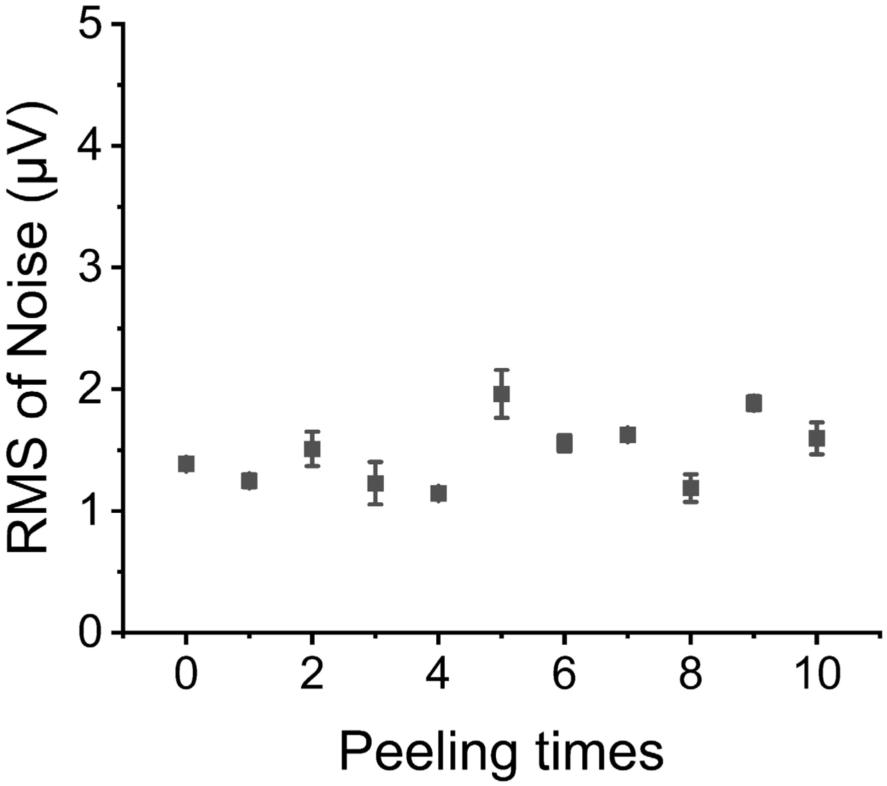

Supplementary Fig. 13 The repetitive test of TPP films on the skin. Each noise after reattachment of TPP film was recorded. n=3 samples for each measurement.

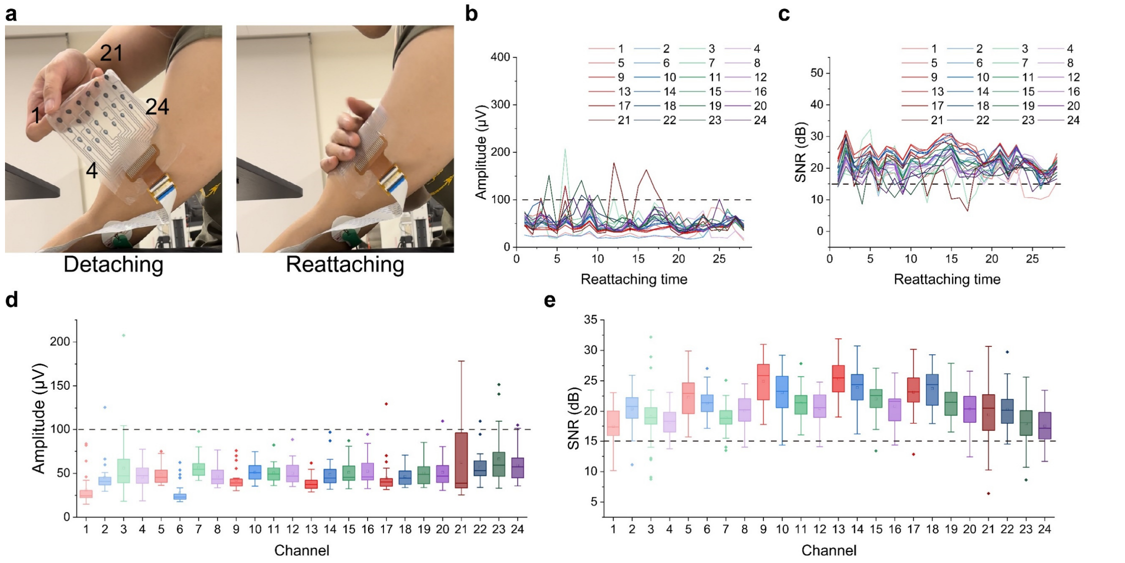

Supplementary Fig. 14 The reattachment test of 24-channel MEAP on the skin.

a Images showing the process of detachment and reattachment of MEAP on biceps brachii.

b, c The baseline noise of each channel and the SNR of each channel plotted for each reattachment. The baseline noise consistently maintained an amplitude of approximately 50 μV across all channels, even after 28 reattachments. Similarly, the SNR remained stable at 20 dB across all channels after 28 reattachments.

d, e Box plots of statistical verification of **b** and **c** for the 28 reattachments. Statistical analysis was conducted to assess the baseline noise and SNR of each channel throughout the reattachment test. 28 reattachments, per channel, were included in the analysis. The box plots depict the mean (centre square), median (centre line), 25th to 75th percentiles (box), and the lower and upper whiskers representing the smallest and largest values that are ≤ 1.5 times the interquartile range, respectively. Outliers are also shown.

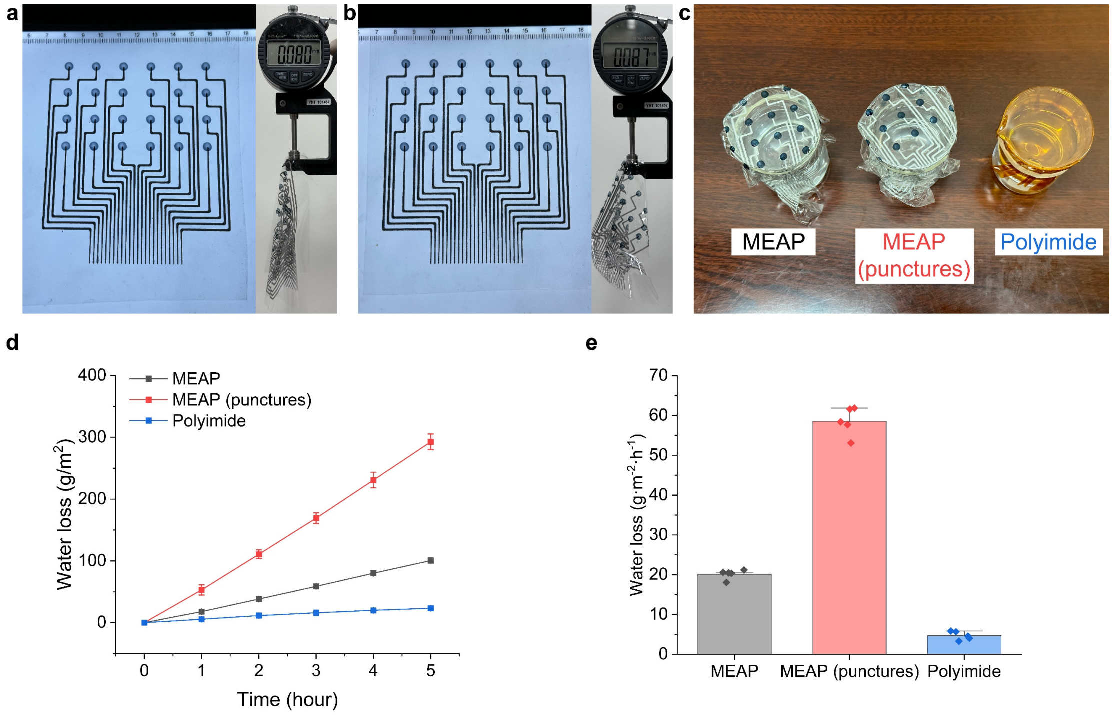

Supplementary Fig. 15 The permeability comparison test between MEAP, MEAP (punctures) and polyimide.

a, b Images showing the MEAP and MEAP (punctures) with thicknesses of 80 and 87 μm, respectively. The MEAP (punctures) features 24 punctures (1 mm in diameter), corresponding to the number of TPP electrodes on the patch.

c The experimental setup for the permeability test. Three beakers, each filled with 100 ml of deionized water, were covered by MEAP, MEAP (punctures), and polyimide, respectively. Each beaker was secured with a rubber band to ensure that water only passed through the cover. The three beakers were placed in a programmable temperature and humidity tester (QHP-360BE, LICHEN, China) set to a temperature of 33 °C and a humidity of 30%, simulating the conditions on human skin.

d The water loss rates in the three beakers. The MEAP (punctures) exhibited higher water loss compared to the MEAP, indicating that the permeability can be adjusted by modifying the physical structure of the substrate. Measurements were recorded for each beaker every hour, with n = 3 samples for each recording.

e The water loss rate of each cover. Considering that the insensible sweat rate of individuals ranges from 12 to 42 g·m^{-2}·h^{-1} [3], the permeability of the MEAP is sufficient to provide a comfortable wearing experience for daily use. Data are presented as mean values ± SD, n=3 samples for each measurement.

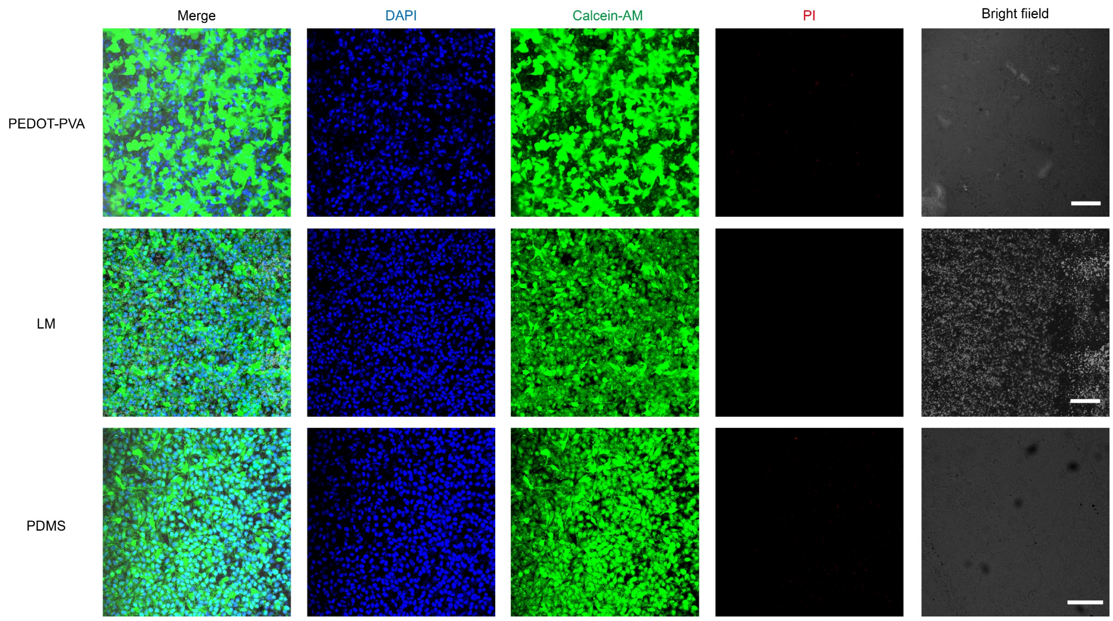

Supplementary Fig. 16 The biocompatibility tests of other materials in the MEAP.

For liquid metal and PDMS, which not directly touching the skin, we also did cell viability tests and results showed all materials have good biocompatibility. Images were collected (x3) independently with similar results.

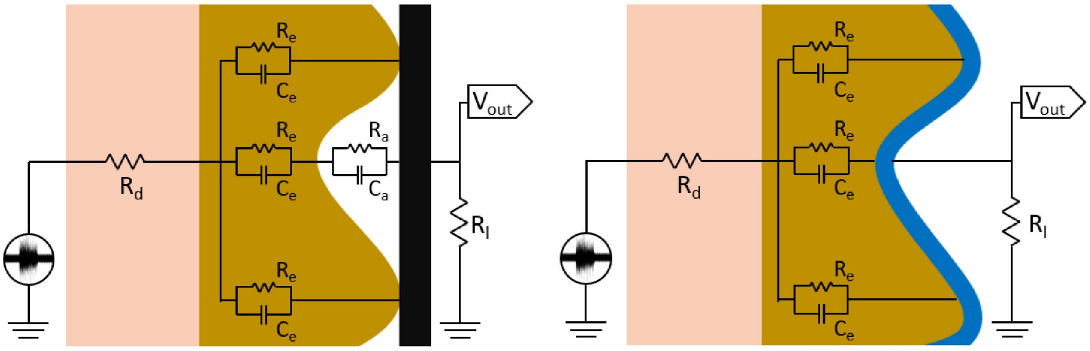

Supplementary Fig. 17 Circuit models of non-conformal and conformal electrodes on skin folds

With the volume of 1 mm^3, the impedance of epidermis is about 157 MΩ, while the impedance of air gap is about 170×10^3 MΩ, which has 3 order-of-magnitude differences[4]. When frontalis muscle contracts, the air gap created by non-conformal electrode provides extra resistance and capacitance to increase impedance of the whole circuit, which decreases signal quality. In addition, the circuit model illustrates how conformal electrodes keep the stable impedance of the whole circuit between skin and electrodes, which help conformal electrodes maintain the signal quality during recording (Fig. 4c). In the circuit model of non-conformal electrodes, dermis resistance (Rd), epidermis resistance (Re) and capacitance (Ce), air gap resistance (Ra) and capacitance (Ca) are considered.

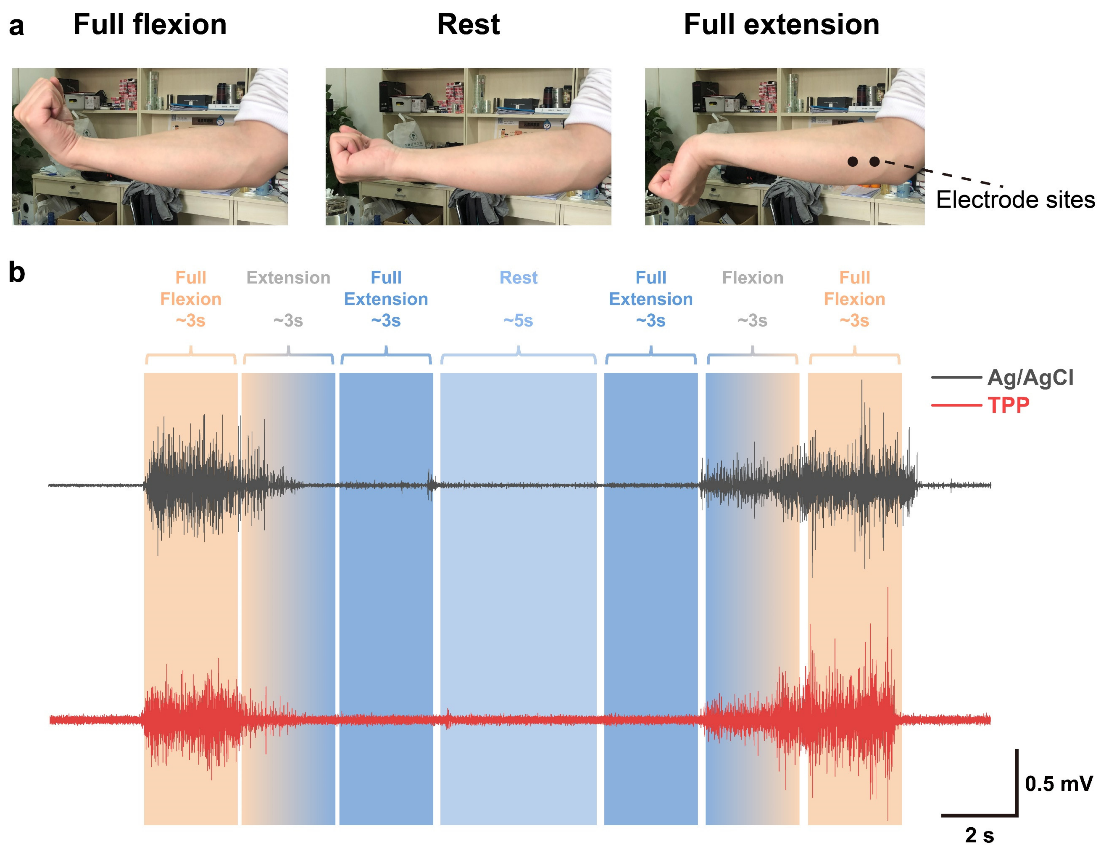

Supplementary Fig. 18 sEMG signals of flexor carpi ulnaris (FCU) recorded by Ag/AgCl and TPP electrodes.

a Photographs of three postures of the wrist driven by FCU and electrode sites on the forearm.

b sEMG signals recorded by Ag/AgCl and TPP electrodes. Both electrodes can record changes of sEMG signals caused by different postures. To record sEMG from exactly same positions, the results were recorded by two trials so time points for each posture do not match perfectly.

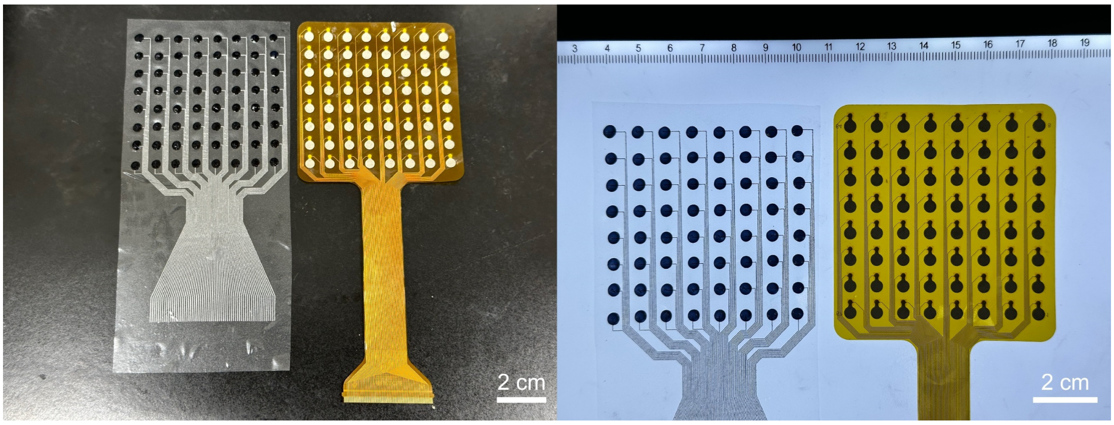

Supplementary Fig. 19 The configuration comparison between MEAP and CA. Both arrays have electrode diameter of 4 mm and IED of 8 mm.

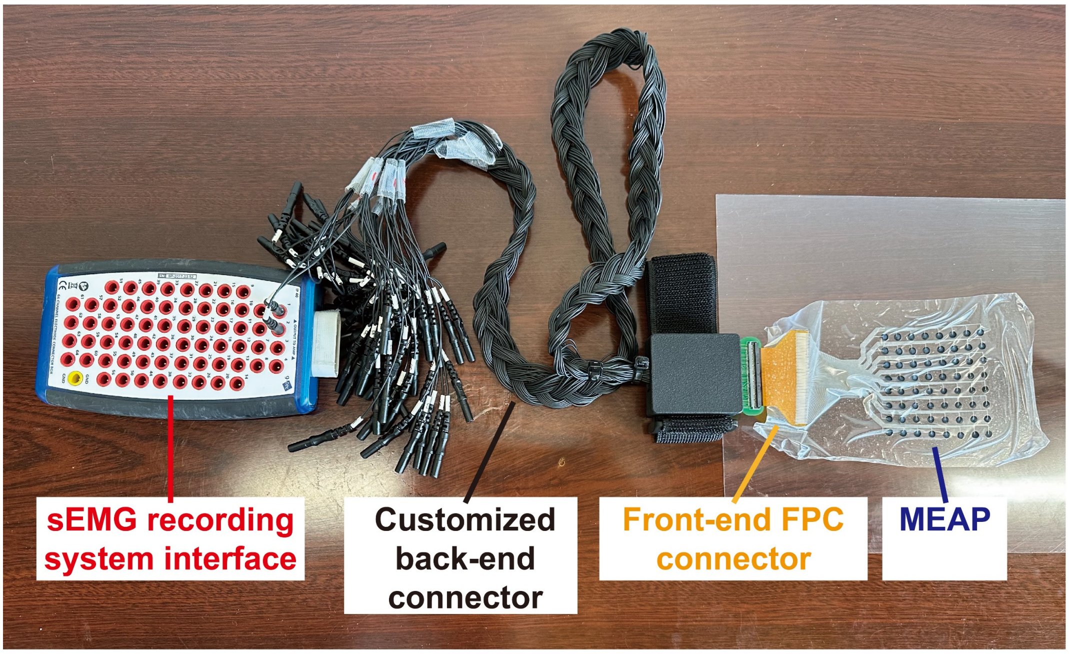

Supplementary Fig. 20 The entire setup for connection between MEAP and EMG recording system. Hot-pressing was employed to combine the Flexible Printed Circuit (FPC) with the MEAP. By using a customized back-end connector, each channel of the MEAP could be independently connected to the G.tec recording system.

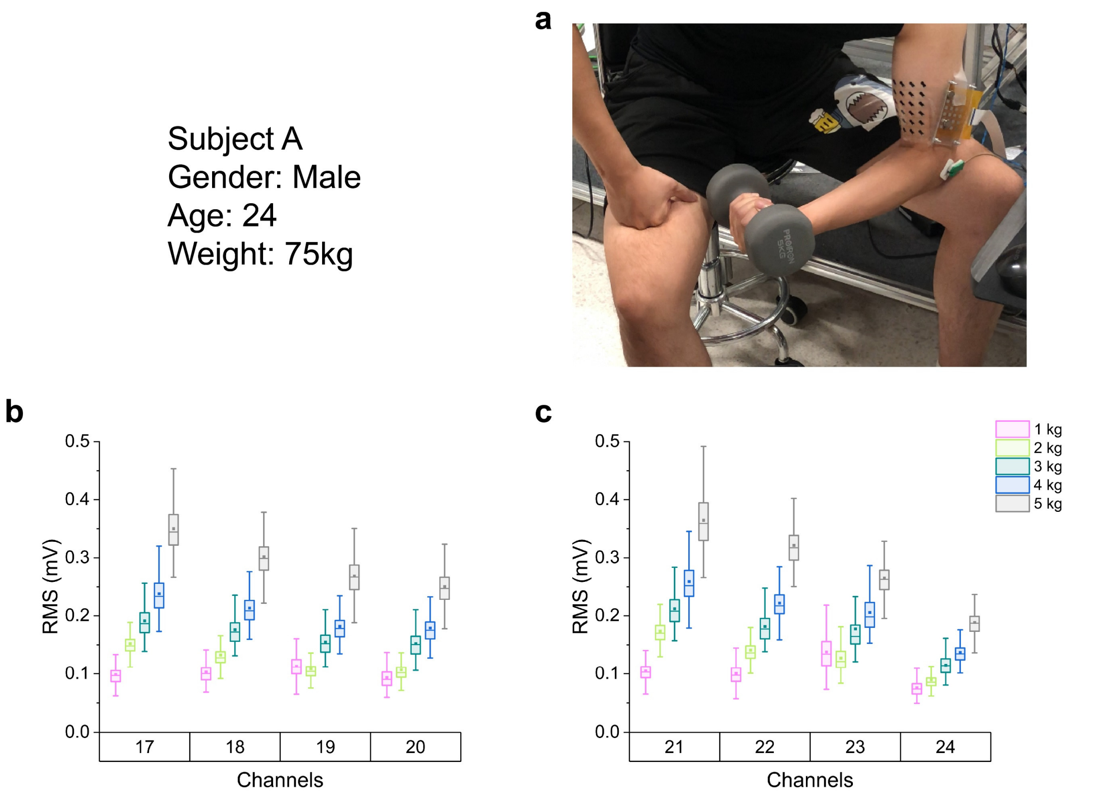

Supplementary Fig. 21 The selection of column of the MEAP on the biceps brachii of subject A.

a A photograph of the subject A during the task.

b, c The RMS values of each channel in the row 5 and 6, respectively, which are closest to the muscle belly of the biceps brachii. Results showed channel 17 and 21 possessed the highest RMS values for most tasks with different loads, which means the first column should be the closest to the muscle belly of biceps brachii. Therefore, the column one is selected for muscle-tendon junction location.

a

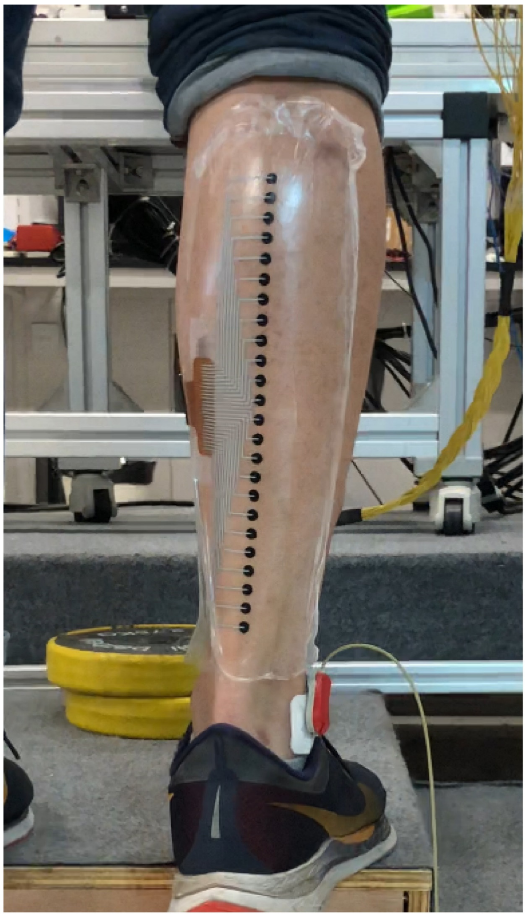

b

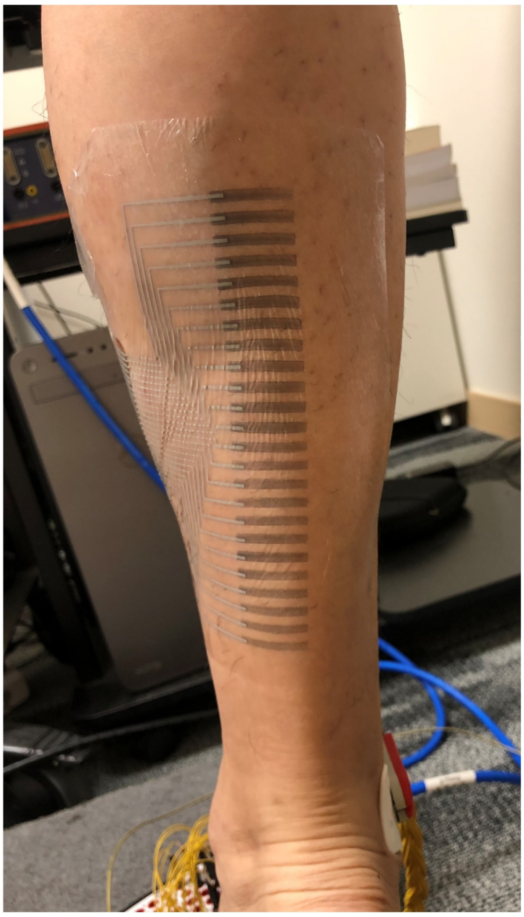

Supplementary Fig. 22 Photographs of MEAPs on the Achilles tendon.

a A photograph of the MEAP #1 with IED of 10 mm, and electrode surface area of 20 mm^2.

b A photograph of the MEAP #2 with IED of 6 mm, and electrode surface area of 30 mm^2.

To figure out where the Achilles tendon junction is on behind the lower leg, we fabricated MEAP #1 with longer IED to explore the position roughly. After the location range is identified, MEAP #2 with shorter IED was attached to conducted finer junction location. Both MEAPs can make junction location monitoring successfully.

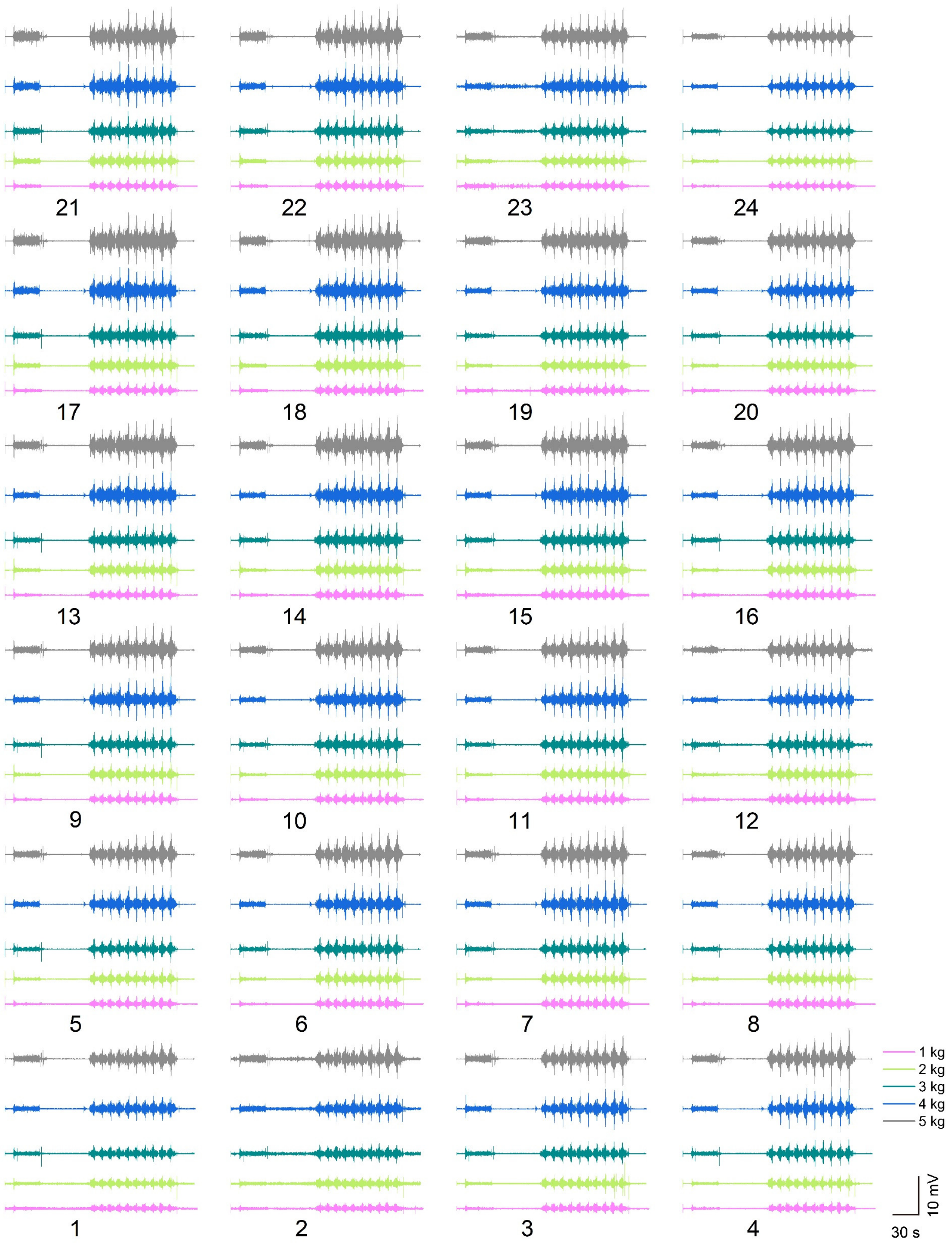

Supplementary Fig. 23 sEMG signals of biceps brachii with five loads recorded by MEAP. All channels successfully record sEMG signals including isometric and dynamic tasks. Differences between each channel and each loading can be distinguished according to excellent signal-to-noise ratio. This makes MEAP become a promising tool for researchers or clinicians to use.

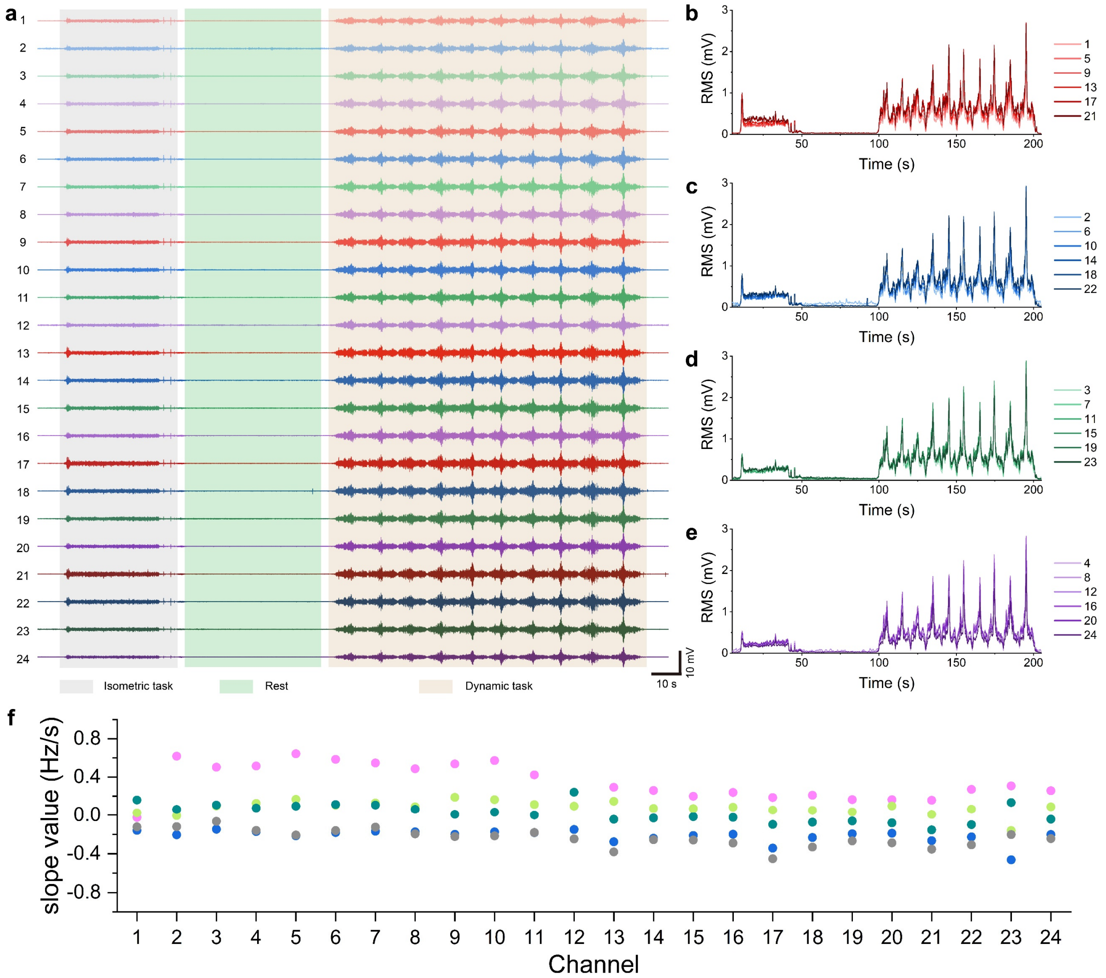

Supplementary Fig. 24 sEMG signals of biceps brachii recorded by MEAP and information derived from them with load of 5 kg.

a sEMG signals recorded by all 24 channels.

b-e RMS values of sEMG signals recorded by each column.

f Slope values of the linear fitting of the sEMG median frequencies during the isometric task recorded by all 24 channels with different loads.

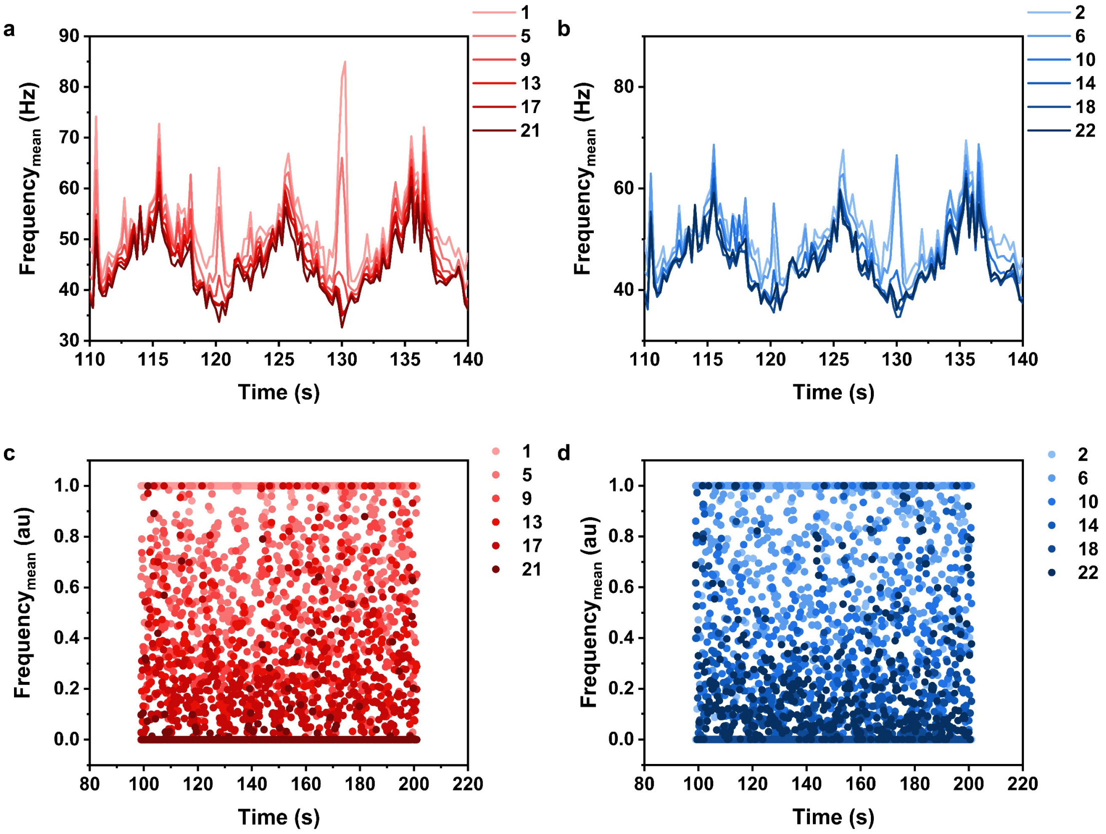

Supplementary Fig. 25 Mean frequencies of sEMG signals with load of 5 kg recorded by MEAP and their normalised values.

a, b Mean frequencies of channels in 1st and 2nd column. Difference in mean frequencies was most obvious when the subject performed full extension during dynamic tasks

c, d Normalised values of mean frequencies during dynamic tasks. Take column 1 as example, the majority of values of channel 21 were normalised to 0 and the majority of values of channel 1 were normalised to 1; and values of channel 17, 13, 9, 5 appeared ascending order in 0~1 range. Few errors can be reduced by averaging in a proper window length.

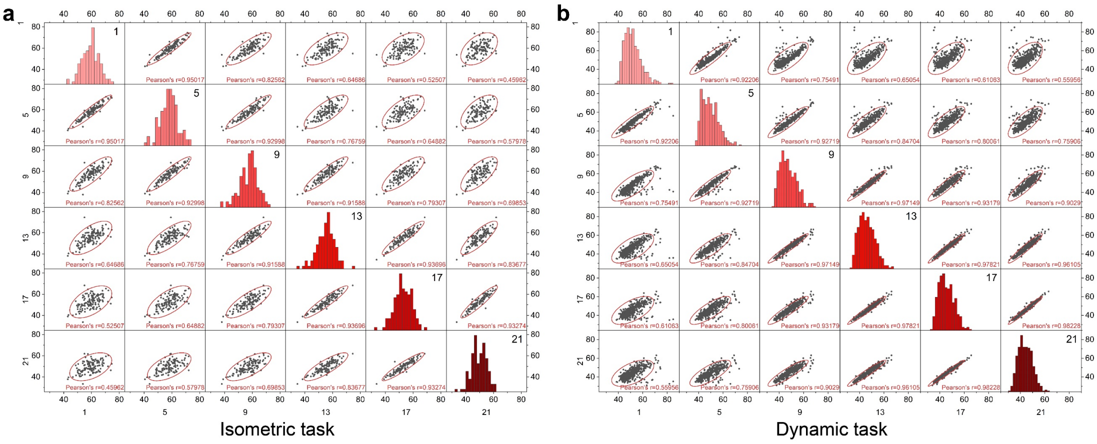

Supplementary Fig. 26 Scatter plot matrix of mean frequencies recorded by 6 channels in column 1.

a Isometric task.

b Dynamic task.

According to Pearson's coefficients, neighbour channels have the highest correlation, but correlation decrease when electrodes' distances between increase. Low correlation between channels on muscle and tendon shows signals can be differentiated to distinguish each part.

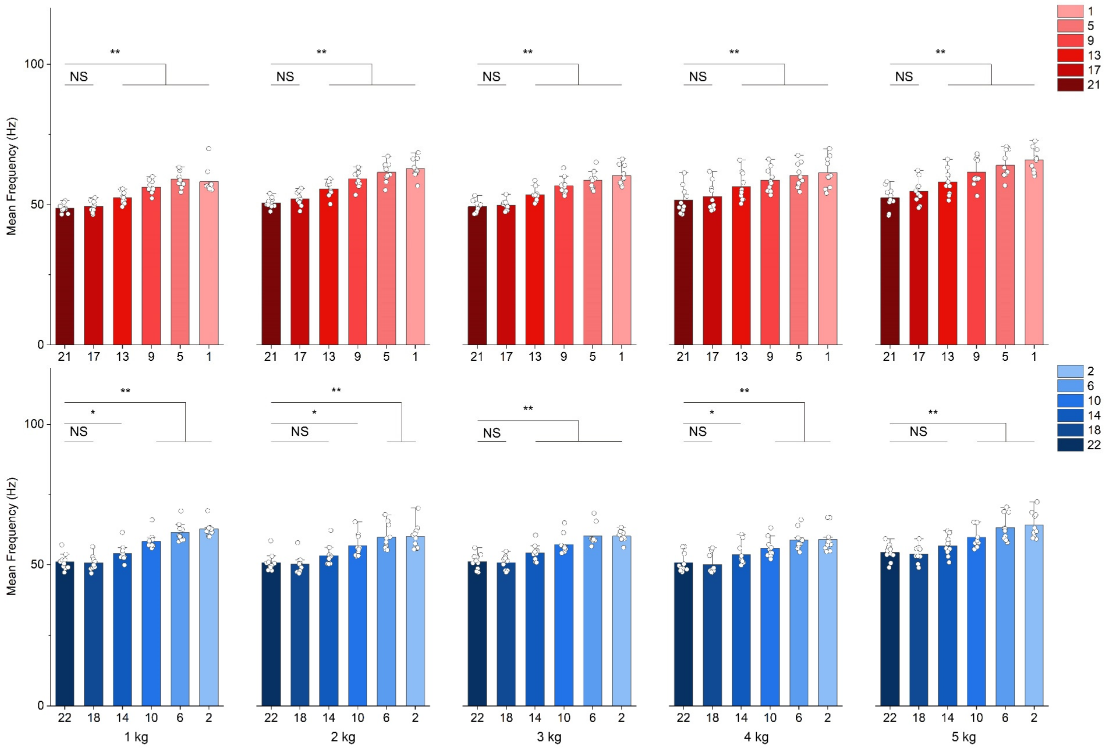

Supplementary Fig. 27 Statistical analysis of the differences in mean frequencies among different channels during full flexions.

In each column, using the most proximal channels (channel 21 and 22) as control, those distal channels all showed significant difference (one-sided t test, *p < 0.05; **p < 0.01). In such cases, muscle-tendon junctions should be longitudinally distinguished between channel 17 and 13 (or channel 18 and 14). n=10 samples for each measurement.

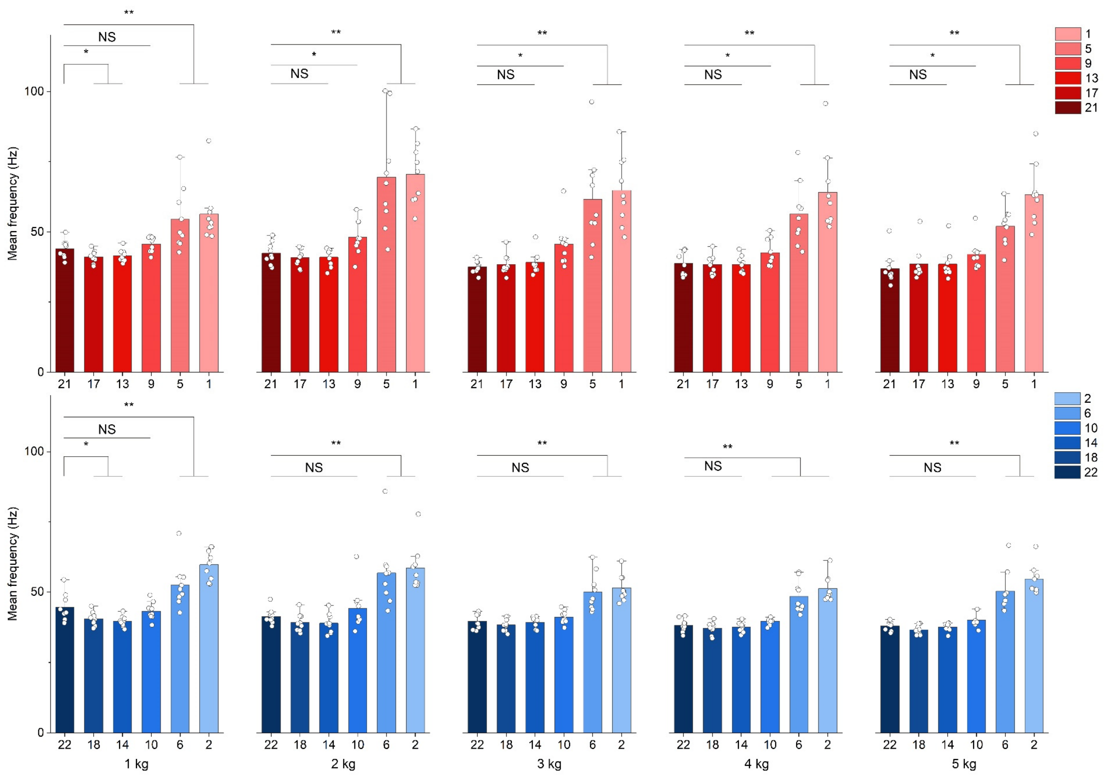

Supplementary Fig. 28 Statistical analysis of the differences in mean frequencies among different channels during full extensions.

In each column, using the most proximal channels (channel 21 and 22) as control, those distal channels all showed significant difference (one-sided t test, *$p < 0.05$; **$p < 0.01$). In such cases, muscle-tendon junctions should be longitudinally distinguished around channel 9 (or between channel 10 and 6), which are closer to elbow compared to full flexions. n=10 samples for each measurement.

Subject B
Gender: Male
Age: 24
Weight: 80kg

Muscle loading
(by RMS)

Muscle Fatigue
(by median frequency)

Muscle-tendon junction location
(by mean frequency)

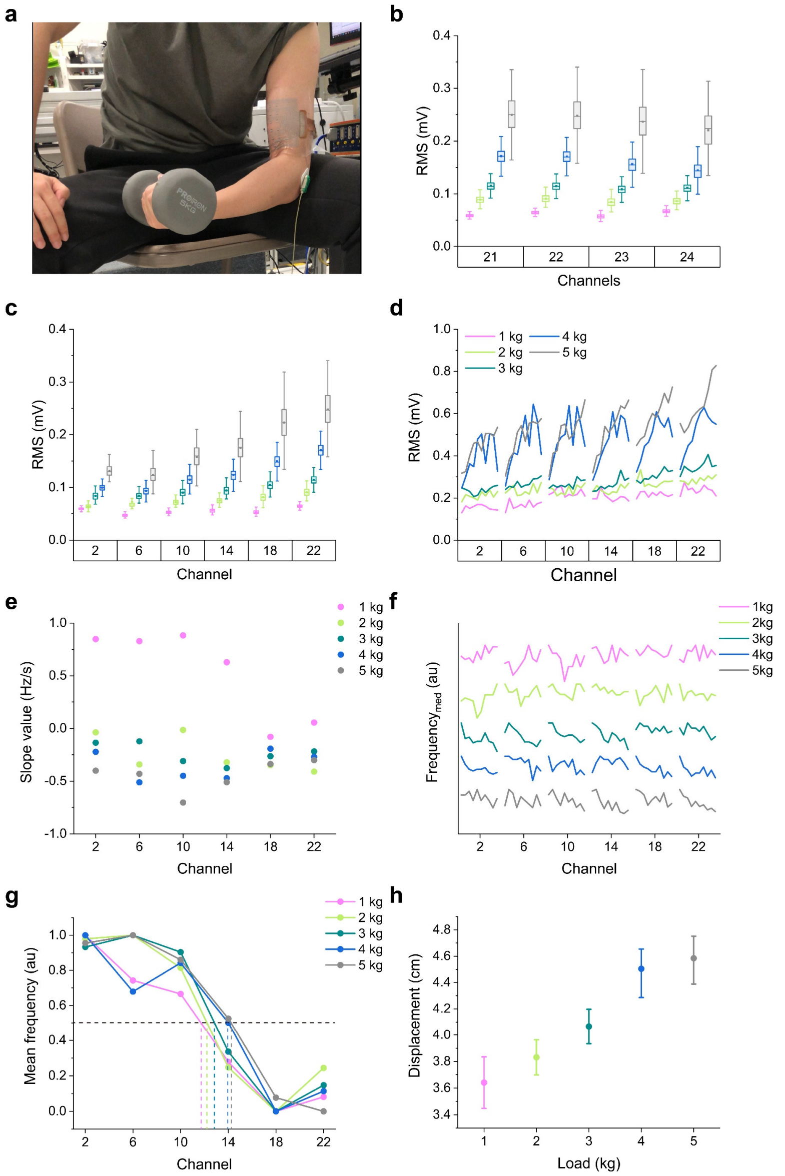

Supplementary Fig. 29 Results of muscle information recorded by MEAP of the task carried by subject B.

a A photograph of subject B during the task.

b RMS results of channels in the last row which is closest to the muscle belly of the biceps brachii. Channel 22 showed the highest RMS value, which therefore, made the second column be selected for identification of different muscle information.

c, d RMS values of sEMG signals in isometric task and in dynamic task, respectively.

e, f Median frequencies of sEMG signals in isometric task and in dynamic task, respectively.

g, h Plots of normalised value of mean frequencies among 6 channels and tendon displacements in the isometric task.

Subject C
Gender: Male
Age: 25
Weight: 60kg

Muscle loading
(by RMS)

Muscle Fatigue
(by median frequency)

Muscle-tendon junction location
(by mean frequency)

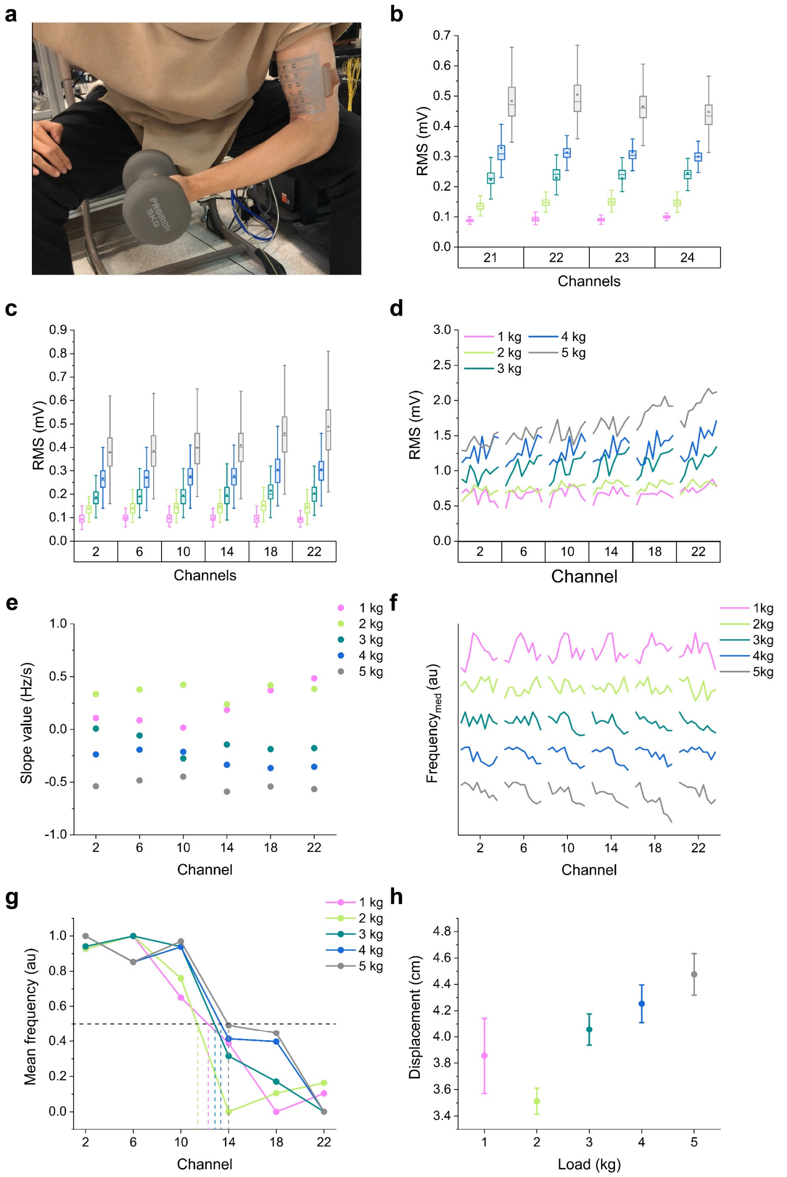

Supplementary Fig. 30 Results of muscle information recorded by MEAP of the task carried by subject C.

a A photograph of subject C during the task.

b RMS results of channels in the last row which is closest to the muscle belly of the biceps brachii. Channel 22 showed the highest RMS value, which therefore, made the second column be selected for identification of different muscle information.

c, d RMS values of sEMG signals in isometric task and in dynamic task, respectively.

e, f Median frequencies of sEMG signals in isometric task and in dynamic task, respectively.

g, h Plots of normalised value of mean frequencies among 6 channels and tendon displacements in the isometric task.

Supplementary Table 1 Comparisons between dry electrodes in other literatures and this work.

	Materials for electrodes	Materials for substrates	Is it intrinsically stretchable?	Strain	The adhesiveness of electrode (N/cm)	The smallest area of the electrode (mm^2)	Electrode-skin impedance at 100 Hz ($K\Omega*cm^2$)	Long-term test (Hour)	RMS of Noise (μV)	Signal-to-noise ratio (dB)	Reference
	Ag	Polyimide	No	80%	0	16.0	12.8	11	N/A	N/A	5
	Ag-filled epoxy	Epoxy	No	N/A	0	100.0	80.0	24	~43.0	16.0	6
	Ag flakes/PDMS	PDMS	Yes	480%	0	100.0	34.0	10	~540.0	N/A	7
	Ag-polytetrafluoroethylene	Polyurethane	Yes	20%	0	600.0	N/A	N/A	~74.0	N/A	8
	Au nanoparticles	Polyimide	No	N/A	0	80.0	N/A	24	~60.0	~21.0	9
	PEDOT:PSS/Glycerol	Silk fiber	Yes	250%	N/A	314.0	~157.0	N/A	N/A	N/A	10
	PEDOT:PSS/Glycerol/Polysorbate	N/A	Yes	100%	0.013	100.0	200.0	12	N/A	35.2	11
	PEDOT:PSS/Polylactic acid	N/A	No	34%	~0.467	176.6	~35.3	N/A	~47.0	22.8	12
	PEDOT:PSS/ Poly(poly(ethylene glycol) methyl ether acrylate)	N/A	Yes	75%	0.005	400.0	N/A	N/A	~60.6	4.5	13
	PEDOT:PSS/Polyvinyl alcohol/Borax	N/A	Yes	400%	N/A	254.3	101.7	N/A	N/A	29.5±1.3	14
	WPU/Deep eutectic solvent/ Tannic acid	N/A	Yes	178%	0.125	1256.0	25	N/A	50.0	~14.0	15
	PEDOT:PSS/Waterborne polyurethane/D-sorbitol	N/A	Yes	43%	0.43	400	15	16	~25	~20	16
	PEDOT:PSS/Polyvinyl alcohol/Tannic Acid	N/A	Yes	54%	0.28	16	100	N/A	11.8	34.96	17
**This work**	**PEDOT:PSS/Polyvinyl alcohol/ Tannic Acid/Liquid metal**	**PDMS**	**Yes**	**188%**	**0.58**	**0.8**	**80**	**120**	**1.0**	**42.3±0.7**	

Supplementary Table 2 Comparisons between sEMG arrays in other literatures and this work.

	Materials for electrodes	Materials for substrates	Is it intrinsically stretchable?	Strain	The adhesiveness of electrode (N/cm)	The number of channels	Success rate of channels	The smallest area of the electrode (mm^2)	Electrode-skin impedance at 100 Hz ($K\Omega*cm^2$)	Long-term test (Hour)	RMS of Noise (μV)	Signal-to-noise ratio (dB)	Reference
	Ag/AgCl ink	Polypropylene	No	N/A	0	16	N/A	5.1	5000.0	N/A	N/A	~24.0	18
	Ag/AgCl ink	Polyethylene terephthalate	No	N/A	0	64	100%	14.5	N/A	2	N/A	~20.0	19
	Ag flakes/PDMS	PDMS	Yes	30%	0	8	100%	26.4	33.0	N/A	N/A	29.5	20
	Ag nanowires	PDMS	Yes	50%	0	18	94.4%	9.6	N/A	N/A	N/A	N/A	21
	Ag nanowires	Thermoplastic polyurethane	Yes	600%	0	4	100%	201.0	1004.8	N/A	~34.0	26.6	22
	Al	Polyethylene terephthalate	No	51%	0	16	100%	84.0	84.0	N/A	~130.0	N/A	23
	Au	Polyimide	No	40%	0	20	N/A	N/A	N/A	N/A	~300.0	~20.0	24
	Au	Polyimide	No	37%	0	64	N/A	0.8	117.0	N/A	~10.0	40.0±8.0	25
	Au	Polyimide	No	N/A	0	64	N/A	3.1	N/A	N/A	N/A	26.0±6.0	25
	Carbon/Silicone rubber	Textile	N/A	N/A	0	14	100%	400.0	320.0	N/A	~100.0	12.8±0.9	26
	MXene	PDMS	No	N/A	0	40	100%	7.1	2.4	N/A	~34.0	N/A	27
	MXene	Parylene-C	No	N/A	0	16	81.25%	2.6	256.0	N/A	~118.0	24.4±1.7	28
	PEDOT:PSS/Cholinium lactate	Kapton	No	N/A	0	16	100%	2.6	45.0	N/A	~40.0	15.6	29
	Stainless steel	Textile	No	N/A	0	150	90.6%	113.0	N/A	N/A	50.6±14.8	30.8±2.4	30
**This work**	**PEDOT:PSS/Polyvinyl alcohol/ Tannic acid/ Liquid metal**	**PDMS**	**Yes**	**188%**	**0.58**	**≥64**	**100%**	**0.8**	**80**	**120**	**1.0**	**42.3±0.7**	

Supplementary References

1. Pailler-Mattei, C., Bec, S. & Zahouani, H. In vivo measurements of the elastic mechanical properties of human skin by indentation tests. *Med. Eng. Phys.* **30**, 599–606 (2008).
2. Li, P., Sun, K. & Ouyang, J. Stretchable and Conductive Polymer Films Prepared by Solution Blending. *ACS Appl. Mater. Interfaces* **7**, 18415–18423 (2015).
3. Zhong, B., Jiang, K., Wang, L. & Shen, G. Wearable Sweat Loss Measuring Devices: From the Role of Sweat Loss to Advanced Mechanisms and Designs. *Adv. Sci.* **9**, (2022).
4. Gabriel, C. *Compilation of the dielectric properties of body tissues at RF and microwave frequencies*. *King's Coll London (United Kingdom) Dept of Physics.* (1996).
5. Harati, A. & Jahanshahi, A. A reliable stretchable dry electrode for monitoring of EEG signals. *Sensors Actuators A Phys.* **326**, 112727 (2021).
6. Alban, M. V., Lee, H., Moon, H. & Yoo, S. Micromolding fabrication of biocompatible dry micro-pyramid array electrodes for wearable biopotential monitoring. *Flex. Print. Electron.* **6**, 045008 (2021).
7. Jiang, Y. *et al.* Flexible and stretchable dry active electrodes with pdms and silver flakes for bio-potentials sensing systems. *IEEE Sens. J.* **21**, 12255–12268 (2021).
8. Yoon, S. *et al.* Highly stretchable metal-polymer hybrid conductors for wearable and self-cleaning sensors. *NPG Asia Mater.* **13**, 4 (2021).
9. Yun, I. *et al.* Stable Bioelectric Signal Acquisition Using an Enlarged Surface-Area Flexible Skin Electrode. *ACS Appl. Electron. Mater.* **3**, 1842–1851 (2021).
10. Li, Q. *et al.* Highly Thermal-Wet Comfortable and Conformal Silk-Based Electrodes for On-Skin Sensors with Sweat Tolerance. *ACS Nano* **15**, 9955–9966 (2021).
11. Tang, W. *et al.* Delamination-Resistant Imperceptible Bioelectrode for Robust Electrophysiological Signals Monitoring. *ACS Mater. Lett.* **3**, 1385–1393 (2021).
12. Won, Y. *et al.* Biocompatible, Transparent, and High-Areal-Coverage Kirigami PEDOT:PSS Electrodes for Electrooculography-Derived Human-Machine Interactions. *ACS Sensors* **6**, 967–975 (2021).
13. Blau, R. *et al.* Intrinsically Stretchable Block Copolymer Based on PEDOT:PSS for Improved Performance in Bioelectronic Applications. *ACS Appl. Mater. Interfaces* **14**, 4823–4835 (2022).
14. Zhou, X. *et al.* Self-healing, stretchable, and highly adhesive hydrogels for epidermal patch electrodes. *Acta Biomater.* **139**, 296–306 (2022).
15. Wang, S. *et al.* Self-adhesive, stretchable, biocompatible, and conductive nonvolatile eutectogels as wearable conformal strain and pressure sensors and biopotential electrodes for precise health monitoring. *ACS Appl. Mater. Interfaces* **13**, 20735–20745 (2021).
16. Zhang, L. *et al.* Fully organic compliant dry electrodes self-adhesive to skin for long-term motion-robust epidermal biopotential monitoring. *Nat. Commun.* **11**, 4683 (2020).
17. Cao, J. *et al.* Stretchable and Self-Adhesive PEDOT:PSS Blend with High Sweat Tolerance as Conformal Biopotential Dry Electrodes. *ACS Appl. Mater. Interfaces* **14**, 39159–39171 (2022).
18. Cantu, E. *et al.* Printed Multi-EMG Electrodes on the 3D Surface of an Orthosis for

Rehabilitation: A Feasibility Study. *IEEE Sens. J.* **21**, 14407–14417 (2021).
19. Moin, A. *et al.* A wearable biosensing system with in-sensor adaptive machine learning for hand gesture recognition. *Nat. Electron.* **4**, 54–63 (2021).
20. Zhu, K., Guo, W., Yang, G., Li, Z. & Wu, H. High-Fidelity Recording of EMG Signals by Multichannel On-Skin Electrode Arrays from Target Muscles for Effective Human-Machine Interfaces. *ACS Appl. Electron. Mater.* **3**, 1350–1358 (2021).
21. Lin, Y. *et al.* High-resolution and large-size stretchable electrodes based on patterned silver nanowires composites. *Nano Res.* **15**, 4590–4598 (2022).
22. Zhao, H. *et al.* Ultrastretchable and Washable Conductive Microtextiles by Coassembly of Silver Nanowires and Elastomeric Microfibers for Epidermal Human-Machine Interfaces. *ACS Mater. Lett.* **3**, 912–920 (2021).
23. Gandla, S. *et al.* Ultrafast Prototyping of Large-Area Stretchable Electronic Systems by Laser Ablation Technique for Controllable Robotic Arm Operations. *IEEE Trans. Ind. Electron.* **69**, 4245–4253 (2022).
24. Mei, Z., Zhao, N., Yang, B. & Liu, J. Flexible Concentric Ring Electrode Array for Low-Noise and Non-Invasive Detection. in *Proceedings of the IEEE International Conference on Micro Electro Mechanical Systems (MEMS)* vols 2021-January 266–269 (Institute of Electrical and Electronics Engineers Inc., 2021).
25. Chandra, S. *et al.* Performance Evaluation of a Wearable Tattoo Electrode Suitable for High-Resolution Surface Electromyogram Recording. *IEEE Trans. Biomed. Eng.* **68**, 1389–1398 (2021).
26. Alizadeh-Meghrazi, M. *et al.* A Mass-Producible Washable Smart Garment with Embedded Textile EMG Electrodes for Control of Myoelectric Prostheses: A Pilot Study. *Sensors* **22**, 666 (2022).
27. Driscoll, N. *et al.* MXene-infused bioelectronic interfaces for multiscale electrophysiology and stimulation. *Sci. Transl. Med.* **13**, eabf8629 (2021).
28. Murphy, B. B. *et al.* A Gel-Free Ti3C2Tx-Based Electrode Array for High-Density, High-Resolution Surface Electromyography. *Adv. Mater. Technol.* **5**, 2000325 (2020).
29. Velasco-Bosom, S. *et al.* Conducting Polymer-Ionic Liquid Electrode Arrays for High-Density Surface Electromyography. *Adv. Healthc. Mater.* **10**, 2100374 (2021).
30. Ting, J. E. *et al.* Sensing and decoding the neural drive to paralyzed muscles during attempted movements of a person with tetraplegia using a sleeve array. *J. Neurophysiol.* **127**, 2104–2118 (2021).
31. Phinyomark, A., Thongpanja, S., Hu, H., Phukpattaranont, P. & Limsakul, C. The usefulness of mean and median frequencies in electromyography analysis. in *Computational Intelligence in Electromyography Analysis - A Perspective on Current Applications and Future Challenges* 195–220 (2012). doi:10.5772/50639.
