# Supplementary material for: Stretchable surface electromyography electrode array patch for tendon location and muscle injury prevention
Source: Nat Commun. 2023 Oct 14;14:6494. doi: 10.1038/s41467-023-42149-x (PMC10576757; doi:10.1038/s41467-023-42149-x)
Supplement: Supplementary file 3 — Description of Additional Supplementary Files [file 41467_2023_42149_MOESM3_ESM.pdf]

Description of Additional Supplementary Files

Supplementary Movie 1: Flexibility and stretchability of MEAP.

Supplementary Movie 2: Commercial array and MEAP on Biceps Brachii.

Supplementary Movie 3: Commercial array and MEAP on muscle-tendon junction.

Supplementary Movie 4: Activation zone movement in the heatmap during sEMG recording on biceps brachii.

Supplementary Movie 5: Demonstration of ultrasound imaging for muscle-tendon junction location.

Supplementary Movie 6: Real-time recording and heatmaps of RMS, median frequencies and mean frequencies of biceps brachii.
