# Supplementary material for: Stretchable surface electromyography electrode array patch for tendon location and muscle injury prevention
Source: Nat Commun. 2023 Oct 14;14:6494. doi: 10.1038/s41467-023-42149-x (PMC10576757; doi:10.1038/s41467-023-42149-x)
Supplement: Supplementary file 10 — Reporting Summary [file 41467_2023_42149_MOESM10_ESM.pdf]

nature portfolio

Corresponding author(s): Samit Chakrabarty, Xingyu Jiang

Last updated by author(s): Sep 14, 2023

# Reporting Summary

Nature Portfolio wishes to improve the reproducibility of the work that we publish. This form provides structure for consistency and transparency in reporting. For further information on Nature Portfolio policies, see our Editorial Policies and the Editorial Policy Checklist.

## Statistics

For all statistical analyses, confirm that the following items are present in the figure legend, table legend, main text, or Methods section.

| n/a | Confirmed | |
|---|---|---|
| ☐ | ☒ | The exact sample size (*n*) for each experimental group/condition, given as a discrete number and unit of measurement |
| ☐ | ☒ | A statement on whether measurements were taken from distinct samples or whether the same sample was measured repeatedly |
| ☐ | ☒ | The statistical test(s) used AND whether they are one- or two-sided<br>*Only common tests should be described solely by name; describe more complex techniques in the Methods section.* |
| ☒ | ☐ | A description of all covariates tested |
| ☒ | ☐ | A description of any assumptions or corrections, such as tests of normality and adjustment for multiple comparisons |
| ☐ | ☒ | A full description of the statistical parameters including central tendency (e.g. means) or other basic estimates (e.g. regression coefficient) AND variation (e.g. standard deviation) or associated estimates of uncertainty (e.g. confidence intervals) |
| ☐ | ☒ | For null hypothesis testing, the test statistic (e.g. *F*, *t*, *r*) with confidence intervals, effect sizes, degrees of freedom and *P* value noted<br>*Give P values as exact values whenever suitable.* |
| ☒ | ☐ | For Bayesian analysis, information on the choice of priors and Markov chain Monte Carlo settings |
| ☒ | ☐ | For hierarchical and complex designs, identification of the appropriate level for tests and full reporting of outcomes |
| ☐ | ☒ | Estimates of effect sizes (e.g. Cohen's *d*, Pearson's *r*), indicating how they were calculated |

*Our web collection on statistics for biologists contains articles on many of the points above.*

## Software and code

Policy information about availability of computer code

| | |
|---|---|
| Data collection | NOVA (Metrohm Autolab, V2.1.4), g.Recorder (g.tec, V5.16.01) |
| Data analysis | Matlab R2020a, NOVA (Metrohm Autolab, V2.1.4), OriginPro 2021, OT BioLab+ (V1.5.9) |

For manuscripts utilizing custom algorithms or software that are central to the research but not yet described in published literature, software must be made available to editors and reviewers. We strongly encourage code deposition in a community repository (e.g. GitHub). See the Nature Portfolio guidelines for submitting code & software for further information.

## Data

Policy information about availability of data

All manuscripts must include a data availability statement. This statement should provide the following information, where applicable:

- Accession codes, unique identifiers, or web links for publicly available datasets
- A description of any restrictions on data availability
- For clinical datasets or third party data, please ensure that the statement adheres to our policy

The authors declare that all data supporting the findings of this study are available within the paper and its Supplementary Information. The sEMG data generated in this study have been deposited in the Science Data Bank database, which is available under restricted access for academic use, access can be obtained by contacting the authors.

## Research involving human participants, their data, or biological material

Policy information about studies with human participants or human data. See also policy information about sex, gender (identity/presentation), and sexual orientation and race, ethnicity and racism.

| | |
|---|---|
| Reporting on sex and gender | All subjects are male in any experiment described in the manuscript or supplementary information. There is no evidence proving that the gender has any effect on any result described in the manuscript or supplementary information. |
| Reporting on race, ethnicity, or other socially relevant groupings | All participants in this study are of Asian descent. Race, ethnicity, or other socially relevant groupings do not impact the findings, as the fundamental electromyography (EMG) remains consistent across individuals. The primary goal of this study is to develop a stretchable sEMG array for continuous muscle activity monitoring, a goal unaffected by factors related to race or ethnicity. |
| Population characteristics | Participants in any experiment described in the manuscript or supplementary information were five healthy male subjects (among them, three were 24 years old, one was 25 years old and one was 20 years old). |
| Recruitment | Participation was fully voluntary, and all subjects provided informed consent before the tests. All subjects were students in Southern University of Science and Technology. There are no selection bias or other biases that may be present and impact results. |
| Ethics oversight | All human experiments were conducted with the approval from the Medical Ethics Committee of Southern University of Science and Technology (approval no. 2021SYG049). A statement of informed consent obtained from the participants was also provided in the manuscript. |

Note that full information on the approval of the study protocol must also be provided in the manuscript.

# Field-specific reporting

Please select the one below that is the best fit for your research. If you are not sure, read the appropriate sections before making your selection.

☒ Life sciences ☐ Behavioural & social sciences ☐ Ecological, evolutionary & environmental sciences

For a reference copy of the document with all sections, see nature.com/documents/nr-reporting-summary-flat.pdf

# Life sciences study design

All studies must disclose on these points even when the disclosure is negative.

| | |
|---|---|
| Sample size | Sample sizes (at least 3) were chosen on the basis of previous experience and statistical standards in the field, which have been clarified in main text for each experiment. Available relevant references: Nat. Commun. 13, 358 (2022); Nat. Commun. 13, 6604 (2022). |
| Data exclusions | No data were excluded. |
| Replication | All attempts at replication were successful. All experiments, including measurements of conductivity, stretchability, impedance, adhesiveness, peeling off test, repeatable usage of array, permeability were replicated three times unless specified. SEM images for the micro-structure of the film and fluorescent images of endothelial cells were each captured three times. Specifically, long-term tests were repeated twice. EMG recordings for muscle-tendon junction location and injury prevention were repeated more than 15 times. |
| Randomization | For all measurements, including conductivity, stretchability, impedance, adhesiveness, peeling-off tests, repeated usage of the array, and permeability, three independent samples were fabricated using the same procedure but from different batches to ensure randomization. In all EMG recording experiments, subjects were recruited randomly from Southern University of Science and Technology, and different arrays were used for repeated tests to ensure randomization. |
| Blinding | The objective of this study is to develop a stretchable sEMG array for continuous muscle activity monitoring. Validation of the TPP electrode's performance is established through comparisons with commercial sEMG electrodes, while the array's validation is based on comparisons with a commercial array. These comparisons rely on objective metrics, such as RMS values and SNR values, making straightforward comparisons of superiority or inferiority.<br><br>For assessments involving tendon displacements and muscle injury, we recruited random participants and followed identical procedures. Data collection adhered to the standards set by SENIAM or CEDE, and data analysis followed specific predefined protocols outlined in the main text. These procedures ensure that anyone else conducting same comparisons, data collection, or data analysis as the authors will yield identical quantified results and arrive at the same conclusions based on these quantifications. As such, blinding procedures were deemed unnecessary. |

# Reporting for specific materials, systems and methods

We require information from authors about some types of materials, experimental systems and methods used in many studies. Here, indicate whether each material, system or method listed is relevant to your study. If you are not sure if a list item applies to your research, read the appropriate section before selecting a response.

## Materials & experimental systems

| n/a | Involved in the study |
| --- | --- |
| ☒ | ☐ Antibodies |
| ☐ | ☒ Eukaryotic cell lines |
| ☒ | ☐ Palaeontology and archaeology |
| ☒ | ☐ Animals and other organisms |
| ☒ | ☐ Clinical data |
| ☒ | ☐ Dual use research of concern |
| ☒ | ☐ Plants |

## Methods

| n/a | Involved in the study |
| --- | --- |
| ☒ | ☐ ChIP-seq |
| ☒ | ☐ Flow cytometry |
| ☒ | ☐ MRI-based neuroimaging |

# Eukaryotic cell lines

Policy information about cell lines and Sex and Gender in Research

| | |
| --- | --- |
| Cell line source(s) | Organism: Homo sapiens. Sex: Female. HumanTissue: umbilical vein/vascular endothelium. |
| Authentication | Karyology performed for one batch of CRL-1730 in 1996 reflected a hypodiploid human cell line with a modal chromosome number of 45 occurring in 72% of the cells counted, all of which had monosomic N13. The rate of polyploid cells among this population was 15.8%. This karyology differed from earlier work-ups performed on the cells that showed approximately 60% of the cells retained 2 chromosomes 13. The apparent clonal variation in cultures of CRL-1730 (most likely dependent upon passage and growth conditions) has also been noted in STR profiles with unstable alleles at D13S317 allele #9, D13S317 allele #11, and D7S820 allele #12. Other coexisting subclones include those with 46,XX,-11,- 13,i(11p),i(11q) and 46,XX,+11,-13 karyotypes. For all karyotypes performed, both X chromosomes appear normal. |
| Mycoplasma contamination | The cell line were not tested for mycoplasma contamination. |
| Commonly misidentified lines (See ICLAC register) | *Name any commonly misidentified cell lines used in the study and provide a rationale for their use.* |
